# Two‐Color Timestamping of Gene Expression with a Chemigenetic Reporter System

**DOI:** 10.1002/cbic.202500494

**Published:** 2025-09-12

**Authors:** Henriette Lämmermann, Jade Nguyen, Juan F. Tamez‐Fernández, Fabien Kuttler, Julien Bortoli Chapalay, Marc Chambon, Gerardo Turcatti, Pablo Rivera‐Fuentes

**Affiliations:** ^1^ Department of Chemistry University of Zurich 8057 Zurich Switzerland; ^2^ Institute of Chemical Sciences and Engineering École Polytechnique Fédérale de Lausanne 1015 Lausanne Switzerland; ^3^ Biomolecular Screening Facility École Polytechnique Fédérale de Lausanne 1015 Lausanne Switzerland

**Keywords:** BiP/GRP78, HaloTag, high‐content screening, reporter gene assays

## Abstract

Stress response pathways rely on coordinated gene expression regulation. In the endoplasmic reticulum, the unfolded protein response plays an important role in maintaining the homeostasis of protein folding and processing. A chemigenetic reporter assay is developed that allows monitoring of BiP/GRP78 expression, a key regulator of the unfolded protein response. The system is based on the coexpression of the self‐labeling protein HaloTag via an internal ribosome entry site. HaloTag allows for flexibility in labeling color as well as labeling timepoint. A two‐color timestamping strategy is designed that provides improved readout sensitivity. The reporter system can be used in live‐cell imaging as well as flow cytometry. It is applied in a high‐content screening experiment in which a new promising activator of BiP/GRP78 expression was identified.

## Graphical abstract

A chemigenetic reporter assay is developed that enables monitoring of BiP/GRP78 expression, an important chaperone and key regulator of the unfolded protein response. The system is based on the coexpression of BiP/GRP78 and the self‐labeling protein HaloTag. Using a two‐color labeling strategy for improved readout sensitivity, the assay is applied in high‐content screening for novel activators of BiP/GRP78 expression.

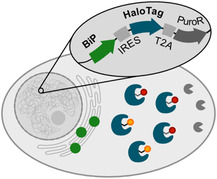

## 1 Introduction

Proper gene expression regulation is crucial for cellular health.^[^ ^1^ ^]^ Stress response pathways rely on coordinated gene expression regulation, and abnormal gene expression has been linked to a range of diseases, including cancer^[^ ^2^ ^]^ and neurodegenerative diseases.^[^ ^3^ ^]^ Therefore, there is a keen interest in elucidating signaling pathways and mechanisms of stress response to discover potential therapeutic targets. Various approaches have been developed to monitor gene expression at different stages of the expression process.

Analysis of the transcriptome can be accomplished with reverse transcription quantitative polymerase chain reaction (RT‐qPCR)^[^ ^4^ ^]^ or, with higher throughput, using microarrays or RNA‐Seq.^[^ ^5^ ^,^ ^6^ ^]^ The expression level of proteins can be analyzed with various methods, such as Western blot,^[^ ^7^ ^]^ immunolabeling,^[^ ^8^ ^]^ or mass spectrometry‐based proteomics.^[^ ^9^ ^]^ To study signaling pathways or dynamic changes, reporter gene assays have been developed. These assays employ luminescence or fluorescence as a detectable signal to measure gene expression or pathway activation, using reporters such as luciferase or fluorescent proteins.^[^ ^10^ ^]^

In these systems, the expression of the reporter is controlled by a promoter or other regulating element that is activated by the pathway of interest. This construct can either be introduced via transient transfection or inserted into the genome. If endogenous promoters are tested, interference from different transcription factors can occur. Additionally, these systems do not reflect the influence of distal regulatory elements or the chromatin environment. To address these limitations, the reporter can be inserted directly downstream of the promoter into the genome. To increase the specificity from a signaling pathway to a specific protein, the reporter expression can be linked directly to the expression of a protein of interest.^[^ ^11^ ^]^ However, the readout by luminescence or fluorescence is not easily adjustable to variable experimental demands.

Here, we describe the development and application of a gene expression reporter system that allows for a flexible fluorescent readout. We used the self‐labeling protein HaloTag as a reporter and incorporated it directly downstream of our gene of interest into the genome. We selected the gene *HSPA5*, which encodes the binding immunoglobulin protein BiP/GRP78, as our gene of interest. BiP/GRP78 plays a crucial role in the endoplasmic reticulum (ER), where it functions as a chaperone and a key mediator in the activation of the unfolded protein response (UPR^ER^).^[^ ^12^ ^]^ Furthermore, BiP/GRP78 is implicated in pathologies such as cancer^[^ ^13^ ^]^ and neurodegenerative diseases.^[^ ^14^ ^]^ Using HaloTag as a reporter, we gain flexibility in labeling color and timepoint. We validate the suitability of the reporter system for live‐cell imaging and flow cytometry. Finally, we demonstrate the advantages of timestamped gene expression monitoring in a high‐content screening (HCS) experiment.

## 2 Results and Discussion

### 2.1 Design of a Chemigenetic Reporter of Gene Expression

**Figure 1:**
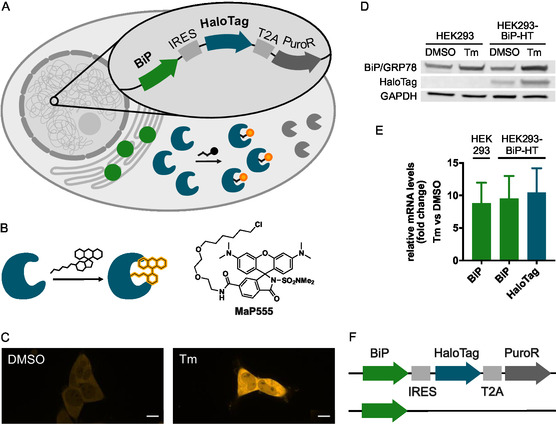
A) HaloTag‐based gene reporter system. Insertion of the sequence IRES‐HaloTag‐T2A‐PuroR into the genome downstream of BiP/GRP78 leads to the coexpression of the protein of interest BiP/GRP78, the reporter HaloTag, and the selection marker puromycin *N*‐acetyltransferase (PuroR) as separate proteins. The expression level of HaloTag, corresponding to the level of BiP/GRP78, can be visualized by fluorescent labeling. B) HaloTag is labeled with fluorogenic ligands, e.g., MaP555. C) Live‐cell fluorescence images (561 nm) of HEK293‐BiP‐HT cells incubated with DMSO (0.1%) or tunicamycin (Tm, 1 µg mL^–1^) for 16 h. HaloTag was visualized by incubation with MaP555 (100 nM, 30 min). Scale bar: 10 µm. D) Western blot of protein lysates from HEK293 or HEK293‐BiP‐HT cells incubated with DMSO (0.1%) or tunicamycin (Tm, 5 µg mL^–1^) for 24 h. E) Relative abundances of HaloTag or BiP/GRP78 mRNA comparing tunicamycin‐treated (Tm, 2 µg mL^–1^, 6 h) with DMSO‐treated (0.1%, 6 h) cells (HEK293 or HEK293‐BiP‐HT) by RT‐qPCR. For each condition, three different samples were prepared, which were each measured in triplicate. Bars represent mean fold change compared to DMSO, error bars represent standard deviation. F) Schematic representation of the two alleles after genome editing.

To implement this reporter gene assay, we incorporated HaloTag into the genome downstream of the gene of interest BiP/GRP78 (**Figure** **1**A). We linked the expression of the gene of interest and HaloTag via an internal ribosome entry site (IRES). The IRES element permits 5′‐cap‐independent translation initiation,^[^ ^15^ ^]^ thus leading to the coexpression of the target and the reporter as two distinct proteins. To enable mutant selection, the insert additionally contained a gene encoding puromycin resistance (puromycin *N*‐acetyltransferase, PuroR). We utilized the self‐cleaving T2A peptide to express HaloTag and PuroR as separate proteins. The insertion of the IRES‐HaloTag‐T2A‐PuroR sequence downstream of the gene of interest thus results in the simultaneous expression of the protein of interest, HaloTag, and puromycin *N*‐acetyltransferase, all as independent proteins. In contrast to fusion protein constructs, this approach avoids any modifications to the protein of interest, BiP/GRP78. We are thus able to monitor BiP/GRP78 with minimal interference with its expression or function.

### 2.2 Establishment and Validation of the Reporter Cell Line

The gene expression reporter cell line was established in HEK293 cells by CRISPR/Cas9 gene editing using the CRISPR/Cas9‐mediated precise integration into target chromosome (CRIS‐PITCh) method.^[^ ^16^ ^]^ The IRES‐HaloTag‐T2A‐PuroR insert was introduced into the 3′‐untranslated region (3′‐UTR) of *HSPA5*. Incorporation of the insert was achieved by transfection with two plasmids, one containing the insert and the other Cas9 along with its guide RNAs (Figure 1, Supporting Information).

**Figure 2:**
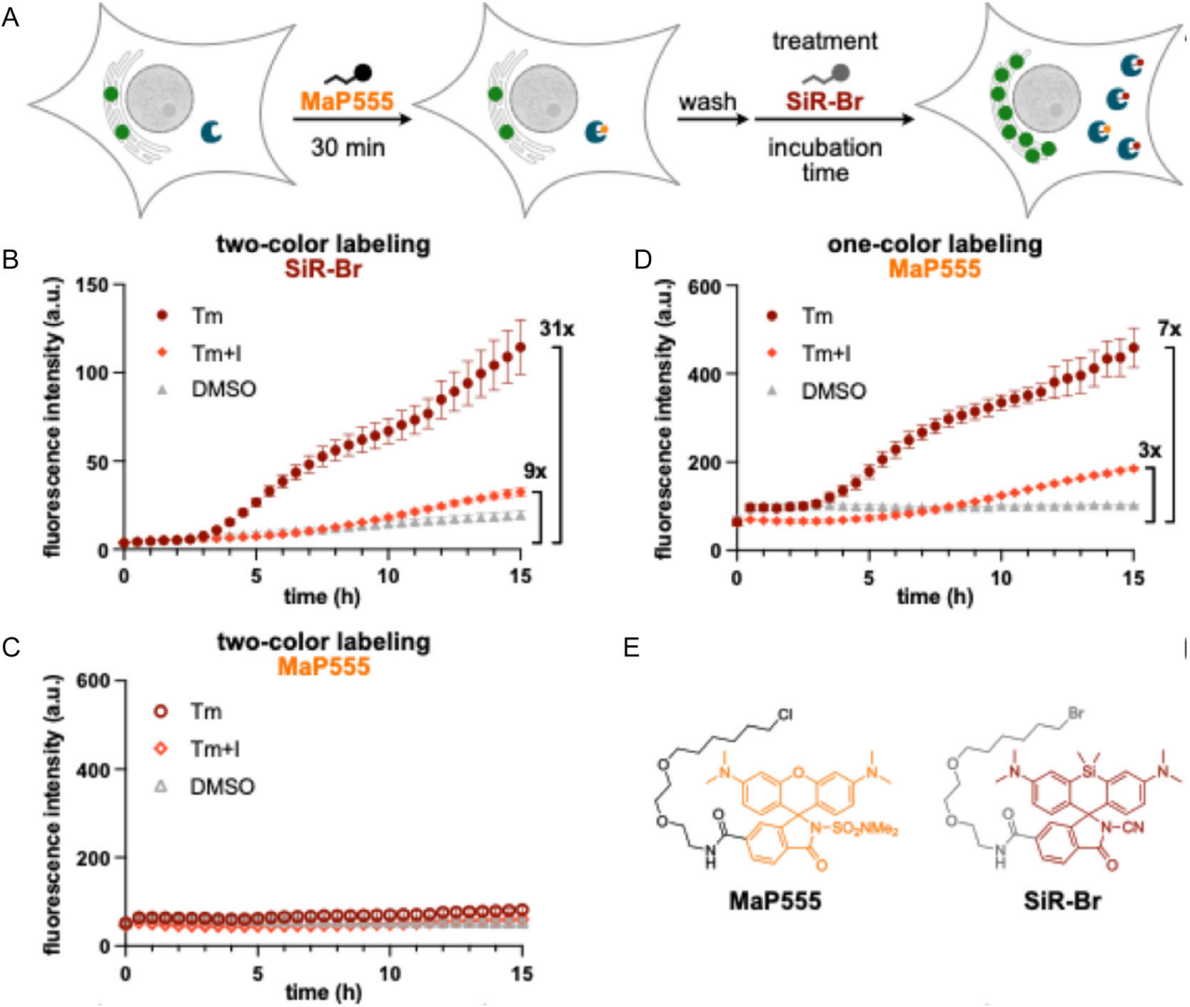
A) Two‐color timestamping of BiP/GRP78 expression. Schematic representation of sequential labeling steps. B–D) HEK293‐BiP‐HT cells were incubated with MaP555 (100 nM, 30 min), washed, and then incubated with three different treatments: DMSO (0.1%), Tm (2 µg mL^–1^), or Tm (2 µg mL^–1^) and PF‐429242 (I, 10 µM), together with MaP555 (100 nM) or SiR‐Br (100 nM). The fluorescence intensity in both channels (561 nm for MaP555 and 640 nm for SiR‐Br) was measured over 15 h. For each condition and channel, intensity values from four different fields of view were plotted. Symbols represent the mean, error bars the standard deviation. Bars on the right indicate the dynamic range. (B) Two‐color labeling, SiR‐Br channel. (C) Two‐color labeling, MaP555 channel. (D) one‐color labeling, MaP555 channel. E) Structures of MaP555 and SiR‐Br.

First, the edited cells were subjected to puromycin selection. Cells from two resistant colonies were labeled with the fluorogenic MaP555‐chloroalkane (MaP555) dye,^[^ ^17^ ^]^ which is only fluorescent when bound to HaloTag (Figure 1B), and sorted into single cells using flow cytometry. The monoclonal cell lines resulting from these single cells were evaluated for incorporation of the gene reporter system by fluorescence microscopy. Cells were incubated with MaP555 and treated with either the known ER stress activator tunicamycin (Tm)^[^ ^18^ ^]^ or DMSO. Tm‐treated cells consistently displayed increased fluorescence intensity compared to DMSO‐treated cells (Figure 1C), indicating that the expression levels of HaloTag increase upon Tm treatment. This result is consistent with the successful incorporation of the insert at the desired position in the genome. We chose the cell line that displayed the strongest response to Tm treatment and further characterized it by Western blot (Figure 1D, Figure 2, Supporting Information), RT‐qPCR, (Figure 1E), and whole‐genome sequencing (WGS, Figure 1F and Table 1, 2, and 3, Supporting Information). We named this cell line HEK293‐BiP‐HT.

The Western blot showed an increase in BiP/GRP78 amount upon incubation with Tm (Figure 1D). This increase was observed for both HEK293 and HEK293‐BiP‐HT cells. In HEK293‐BiP‐HT cells, HaloTag and its increase in Tm‐treated cells could be detected (Figure 1D). Consistent with the results of fluorescence microscopy, Tm‐treated cells displayed higher expression levels of BiP/GRP78 and HaloTag than DMSO‐treated cells (Figure 1D). For visualization by Western blot, incubation with a higher concentration of Tm compared to the microscopy experiments was necessary to be able to observe a difference between DMSO‐ and Tm‐treated cells. This discrepancy between microscopy and Western blot can be explained by the higher sensitivity of fluorescence microscopy.

At the transcriptional level, the relative increase in abundance of HaloTag and BiP/GRP78 mRNA in Tm‐compared to DMSO‐treated cells was measured in HEK293 and HEK293‐BiP‐HT cells (Figure 1E). The amount of BiP/GRP78 mRNA increased to a similar extent in HEK293 and HEK293‐BiP‐HT cells, indicating that genome editing did not affect transcription efficiency. Additionally, both BiP and HaloTag mRNA increased by a similar factor upon Tm treatment of HEK293‐BiP‐HT cells (Figure 1E). This result confirms that transcription of HaloTag increases proportionally to that of BiP/GRP78 when activated by Tm treatment, consistent with the fact that BiP/GRP78 and HaloTag are encoded on the same transcript. However, to directly correlate fluorescence intensity with mRNA levels, the translation efficiency and turnover of the BiP/GRP78 mRNA, as well as the IRES efficiency and turn‐on of the fluorophore would need to be considered as well.

Finally, WGS was performed to confirm the insertion of IRES‐HaloTag‐T2A‐PuroR at the desired position in the 3′‐UTR of the *HSPA5* gene and to exclude any off‐target insertions (Figure 1F and Table 1, Supporting Information). WGS revealed successful gene editing of one allele. Furthermore, no off‐target insertions were detected, confirming that HaloTag expression is solely dependent on BiP/GRP78 expression (for a discussion of other minor mutations found by WGS, see Tables 2 and 3, Supporting Information). On the one hand, the heterozygosity of the HEK293‐BiP‐HT cell line results in less HaloTag available for labeling. Nonetheless, we observe a strong fluorescent signal after labeling with fluorogenic dyes. On the other hand, this heterozygosity also presents the advantage that one allele remains almost entirely unmodified (except for a small deletion at the cut site resulting from nonhomologous end joining).

### 2.3 Two‐Color Timestamping of BiP/GRP78 Expression in HEK293‐BiP‐HT Cells

Although we were able to distinguish between cells exposed to Tm and cells exposed to DMSO using only one fluorogenic dye, we reasoned that we could monitor subtler changes in gene expression in a timestamping experiment with two fluorogenic dyes of different colors. This is especially relevant when the protein of interest is expressed at a high level under basal conditions, which makes it difficult to detect small changes in expression because the basal fluorescence intensity is already high. This is the case for BiP/GRP78, which is present in concentrations of ≈0.5 mM in the ER.^[^ ^19^ ^]^ Moreover, being able to distinguish between expression levels before and after stimulation would be useful while studying genes that display large variations in expression levels from cell to cell. Thus, dual‐color timestamping of gene expression is useful in various scenarios.

**Figure 3:**
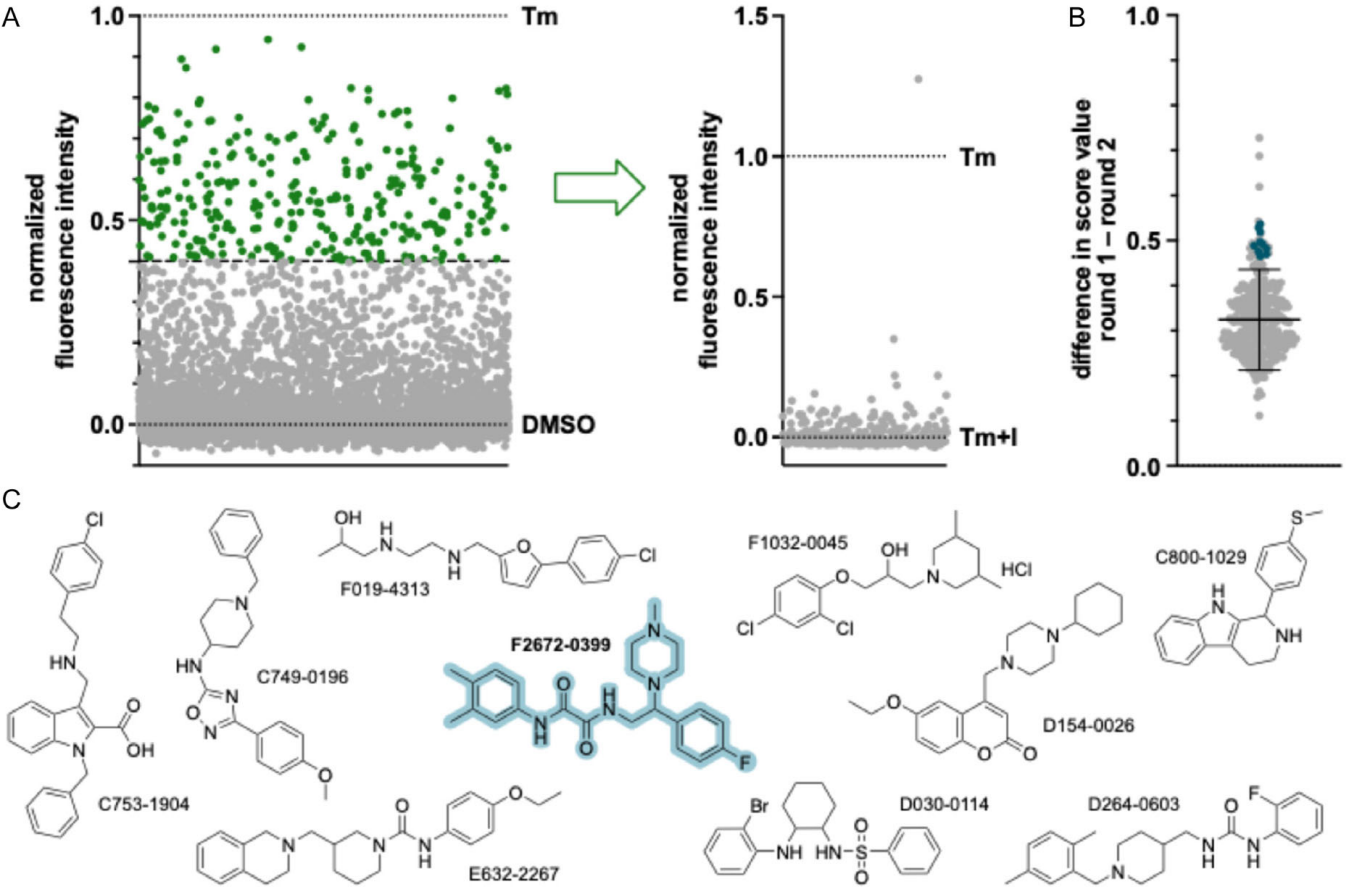
A) High‐content screening for BiP/GRP78 activators. Both screening rounds; first screening for BiP/GRP78 activation (left), then for activation in the presence of the inhibitor PF‐429242 (right). In the first round, HEK293‐BiP‐HT cells were incubated with MaP555 (100 nM, 30 min), washed, then incubated with 7680 screening compounds (10 µM) and SiR‐Br (100 nM) for 24 h. Plotted are the normalized values of all compounds, ≥0.40 in green, <0.40 in gray (DSMO = 0, tunicamycin (Tm) = 1). The 320 selected compounds were used in the second round, where the incubation procedure of the first round was repeated in the presence of PF‐429242 (I, 10 µM). Plotted are the average normalized values of all compounds (Tm + I = 0, Tm = 1). B) Difference in score values between screening rounds 1 and 2, selected hits in blue. Black lines indicate mean and standard deviation. C) Structures of selected hits (blue dots in **B**).

We envisioned the following workflow (**Figure** **2**A): Prior to stimulating the cells to express BiP/GRP78, the existing HaloTag protein, which correlates with the basal expression level of BiP/GRP78, is labeled first. Probe MaP555 was chosen for this purpose because it can label HaloTag quantitatively and with fast kinetics (Figure 3, Supporting Information).^[^ ^17^ ^]^ Subsequently, the excess unbound dye is removed in a washing step, followed by treatment of the cells with an inducer of BiP/GRP78 expression and another fluorogenic dye. For this second step, we chose to use a silicon rhodamine cyanamide derivative functionalized with a bromoalkane (SiR‐Br) for fast HaloTag labeling (Figure 2E, Figure 3, Supporting Information).^[^ ^20^ ^]^ These two dyes exhibit low background fluorescence and absorb in the yellow and red parts of the visible spectrum, ensuring low phototoxicity, while being recorded in two different imaging channels without bleed‐through (Figures 4 and 5, Supporting Information). Thus, this experiment allows the observation of basal and induced (timestamped) expression levels of BiP/GRP78 at the same time for each individual cell.

We applied this workflow to monitor the expression of BiP/GRP78 after treatment with Tm (Figures 2B and C, Video 1, Supporting Information). Prior to exposure to Tm, cells were labeled with MaP555 and washed. MaP555 fluorescence intensity remained unchanged after treatment with Tm, indicating that the washing step was efficient (Figure 2C). In contrast, the fluorescence intensity of SiR‐Br, which was added to the cells with Tm and remained in the growth medium, increased significantly over time, confirming that the second color can be used to monitor changes in BiP/GRP78 overexpression above basal levels upon stimulation (Figure 2B and Video 1, Supporting Information). The fluorescence of SiR‐Br increased significantly less in cells treated only with DMSO. This smaller increase reflects the continuous, natural expression levels of BiP/GRP78 in unstimulated cells.

Tm inhibits *N*‐linked glycosylation,^[^ ^21^ ^]^ activating the UPR^ER^, and expression of BiP/GRP78 is primarily regulated by the ATF6 branch of the UPR^ER^.^[^ ^22^ ^]^ To demonstrate that HaloTag is also expressed through this pathway, we incubated HEK293‐BiP‐HT cells with Tm in the presence of an inhibitor of the ATF6 pathway—the site‐1 protease (S1P)‐inhibitor PF‐429242.^[^ ^23^ ^]^ The fluorescence increase of SiR‐Br was reduced significantly compared to the increase observed with Tm (Figure 2B). This experiment further confirms that our reporter system can be used to monitor changes in the expression levels of BiP/GRP78. Additionally, we verified that the incubation procedure does not induce any upregulation in BiP/GRP78 and HaloTag levels (Figure 6, Supporting Information).

The maximum expression level of BiP/GRP78 that we could measure was limited by cell death following treatment with Tm (Figure 2B and D −15 h). Using this value as the maximum, we determined a dynamic range of 31 for two‐color imaging, whereas single‐color imaging gave a dynamic range of only 7. The larger dynamic range of the two‐color imaging strategy reflects the fact that after blocking the HaloTag proteins that were already present prior to stimulation, the measurement starts from a near‐zero baseline. Therefore, even weak BiP/GRP78 overexpression, such as that observed upon treatment of cells with Tm and PF 429242, gives a strong signal (ninefold increase). In contrast, one‐color monitoring always starts at a high baseline because of the existing HaloTag in the cell, and therefore, the weak overexpression of BiP/GRP78 upon stimulation with Tm and PF‐429242 gives a much weaker signal (threefold increase). These results demonstrate that two‐color imaging provides better sensitivity than one‐color imaging.

To enable the analysis of a large number of cells, we furthermore validated our method for use in flow cytometry. Using the two‐color labeling strategy, cell populations treated with DMSO, Tm + PF‐429242, and Tm could be readily distinguished (Figure 7, Supporting Information). Furthermore, flow cytometry enables straightforward analysis of the correlation between basal and induced expression levels at the single‐cell level (Figure 7, Supporting Information).

### 2.4 High‐Content Screening with Timestamping

BiP/GRP78 is a chaperone that promotes protein folding in the ER.^[^ ^24^ ^]^ As such, its overexpression has drawn attention as a potential strategy to alleviate the toxicity of secreted misfolded proteins.^[^ ^25^ ^]^ In the context of Parkinson's disease, recent experiments in patient‐derived midbrain cultures demonstrated that increased ER protein folding capacity alleviates *α*‐synuclein neurotoxicity.^[^ ^26^ ^]^ Furthermore, overexpression of BiP/GRP78 in nigral dopamine neurons of a rat model of Parkinson's disease significantly decreased the neurotoxicity of secreted *α*‐synuclein.^[^ ^27^ ^]^ These results suggest that small molecules that could induce overexpression of BiP/GRP78 may be of potential therapeutic value in protein misfolding pathologies.

Gene reporter assays are particularly useful in high‐throughput screening experiments in which large libraries of compounds are tested for biological activity. As our assay allows monitoring of subtle changes, we chose an image‐based, HCS readout using an automated fluorescence microscope coupled with an automated image analysis pipeline instead of the classical bulk fluorescence readout using a standard plate fluorimeter. We thus set out to screen a diverse library of small molecules to find activators of BiP/GRP78 overexpression using our two‐color timestamping assay in HEK293‐BiP‐HT cells. As BiP/GRP78 expression is induced by the ATF6 pathway of the UPR^ER^, we anticipated that many molecules with general ER toxicity could be misidentified as hits. To obtain a more refined list of hits, we planned to carry out counter‐screening in the presence of the inhibitor PF‐429242. We envisioned that this workflow would enable us to identify compounds that activate BiP/GRP78 specifically through the ATF6 pathway without inducing general ER stress.

For both screening rounds, the applicability of the two‐color timestamping assay to HCS was assessed and validated using the Z’ factor, which is a measure of data variation and dynamic range (see Supporting Information).^[^ ^28^ ^]^ For the first round, DMSO and Tm were selected as the negative and positive controls, respectively. For the second round, Tm together with PF‐429242 was chosen as the negative control, with Tm as the positive control. In initial validation experiments, the Z’ factor was 0.92 and 0.71 for the first and the second rounds, respectively, validating the robustness of our two‐color assay in HEK293‐BiP‐HT cells for use in HCS.

### 2.5 High‐Content Screening for Small‐Molecule Activators of BiP/GRP78 Expression

We selected a library consisting of chemically diverse compounds for our screening, as we wanted to maximize the chemical space we explored during screening. The first round of screening (**Figure** **3**A) was conducted with 7680 compounds in duplicates from the first layer of the chemically diverse collection (CDC) from the biomolecular screening facility (BSF) at EPFL. We evaluated the activity of the compounds with a score value calculated as a relative measure of activation based on normalized controls (DMSO = 0, Tm = 1). In the first round (primary screen), many compounds exhibited a normalized value > 0. Of these molecules, 320 (4% of 7680) compounds exhibited a score value of ≥0.40 and were thus selected as hits. In a repetition of the first round, 205 of these 320 hits were confirmed.

We subjected all 320 selected hit compounds from the first screening round to the counter‐screen (Figure 3A). For this screening in the presence of the ATF6 pathway inhibitor PF‐429242, we defined a new score value based on the controls Tm + I and Tm. Most of the 320 hit compounds exhibited low score values in the second screening round. We selected the molecules with the highest difference in score values between the first and second screening rounds (Figure 3B). We filtered these compounds based on published records of biological activity and precipitate or other artifacts in the microscopy images. Out of the 20 molecules with a difference > 0.46 in score values between the first and second rounds, half were excluded either because they had been reported as active structures in other screening campaigns or due to visible precipitate in the fields of view acquired during our screening rounds. We thus identified 10 compounds with diverse structures as new promising activators of BiP/GRP78 (Figure 3C). By RT‐qPCR, we could successfully validate one of these hits: compound F2762‐0399. We observed a twofold increase in BiP/GRP78 and HaloTag mRNA upon incubation with F2672‐0399 (Figure 8, Supporting Information). To the best of our knowledge, this compound has not been previously identified as an activator of BiP/GRP78 overexpression or as a general ER stressor.

### 2.6 High‐Content Screening Highlights the Advantage of Two‐Color Labeling Strategy

To evaluate the impact of using the two‐color labeling strategy on the outcome of the screening experiment, we compared our two‐color to a “classical” one‐color labeling method. We simulated one‐color labeling by using the same dye (MaP555) for both labeling steps.

First, we compared the Z’ factors of the different experiments. For one‐color labeling, the Z’ factors were calculated to be 0.91 for the first round (−inhibitor) and 0.84 for the second round (+inhibitor). These values are very similar to or even higher than those of the two‐color experiment. Thus, the quality of the assay is not compromised by the one‐color labeling approach. However, the number of hits we would have found with one‐color labeling was drastically reduced compared to the number of hits we discovered using our labeling strategy. In the first round, only 59 of the 320 hits (18%) or 43 of the confirmed hits (21%) would have been found using one‐color labeling. Of the ten selected hits, only two would have been discovered with the one‐color strategy. Compound F2762‐0399, validated by RT‐qPCR, is one of these hits that would have been overlooked in the one‐color readout (Figures 9, 10, and 11, Supporting Information), but could be discovered by two‐color timestamped monitoring of BiP/GRP78 overexpression. This comparison highlights the sensitivity of our system compared to other commonly used assays.

The difference between validation and screening emphasizes that our labeling strategy excels in detecting small changes in gene expression. For strong—and often toxic—activators such as Tm, a single‐color readout is sufficient. However, for lead compounds—in particular those that do not induce general ER stress and toxicity—we do not anticipate changes in gene expression that are as drastic as for Tm. To observe such subtle changes, the two‐color timestamped readout is crucial.

## 3 Conclusions

We developed a gene expression reporter system based on the coexpression of the protein of interest BiP/GRP78 and the reporter HaloTag. The system was established as a stable cell line by the incorporation of the reporter downstream of the gene of interest into the genome, resulting in a direct correlation between the amount of BiP and HaloTag. We devised a two‐color timestamping labeling strategy that enables us to detect subtle changes in expression by blocking the intrinsic fluorescence background generated by basal expression levels, making it easier to monitor changes exclusively after stimulating the cell.

The method can be used in live‐cell imaging and flow cytometry. The compatibility of the method with the latter technique suggests that it could be adapted to sort cells based on their gene expression levels for downstream genetic analyses. Such studies could, e.g., help characterize the genetic background of cells that are overly sensitive or resistant to upregulation of genes of interest. Furthermore, we demonstrated the value of the system in HCS. With our approach, we were able to find an activator of BiP/GRP78 expression that would have been otherwise overlooked.

Whereas the concept of using IRES in combination with a reporter protein is not new,^[^ ^11^ ^]^ our method has unique advantages over existing solutions. Other BiP/GRP78 reporter systems have employed the expression of luciferase under the control of the BiP/GRP78 promoter or the expression of a mGFP‐fused BiP/GRP78.^[^ ^29^, ^30^, ^31^ ^]^ In these strategies, either the promoter is not in its native genomic environment or the protein function might be compromised by the fusion. In the context of these existing reporter systems for BiP/GRP78, our design offers the advantage of minimal disruption of BiP/GRP78 as well as the investigation of the promoter in its genomic context.

Compared to reporter systems using luciferase or fluorescent proteins to generate a luminescence or fluorescence readout,^[^ ^11^ ^]^ our system offers the advantage that the readout color can be changed easily by exchanging the HaloTag dyes. However, the two‐color labeling strategy involves the need for two additional labeling and wash steps, which might compromise throughput in very large screens. Nevertheless, we were able to perform a screening experiment by labeling the reporter cells in batch before cell seeding to enable high throughput. This solution might apply to many other studies as well. Furthermore, our gene expression reporter system can, in principle, be adapted easily to any target whose activity is regulated by expression. To change the target protein, only two modifications of the plasmids used for gene editing are necessary, which can be accomplished in two easy cloning steps.

## Conflict of Interest

The authors declare no conflict of interest.

## Author Contributions

**Henriette Lämmermann**, **Jade Nguyen**, and **Pablo Rivera‐Fuentes**: conceptualized the project; **Henriette Lämmermann** and **Jade Nguyen**: performed cloning, cell culture, FACS, and imaging experiments; **Henriette Lämmermann**: performed validation experiments; **Henriette Lämmermann**, **Jade Nguyen**, **Fabien Kuttler**, **Gerardo Turcatti**, and **Pablo Rivera‐Fuentes**: planned high‐content screening experiments; **Henriette Lämmermann**, **Fabien Kuttler**, **Julien Bortoli Chapalay**, and **Marc Chambon**: performed screening experiments; **Juan F. Tamez‐Fernández**: contributed to the synthesis of small‐molecule hits. All authors analyzed data; **Henriette Lämmermann** and **Pablo Rivera‐Fuentes**: wrote the manuscript with feedback from all authors; **Gerardo Turcatti**: supervised high‐content screening experiments; **Pablo Rivera‐Fuentes**: acquired funds and supervised the project.

## Supporting information

Supplementary Material

## Data Availability

All data supporting this paper are available through Zenodo (DOI: 10.5281/zenodo.15274965).
