# Supplementary material for: Two‐Color Timestamping of Gene Expression with a Chemigenetic Reporter System
Source: Chembiochem. 2025 Sep 12;26(19):e202500494. doi: 10.1002/cbic.202500494 (PMC12498184; doi:10.1002/cbic.202500494)
Supplement: Supplementary file 1 — Supplementary Material [file CBIC-26-e202500494-s001.zip › cbic202500494-sup-0001-SuppData-S1.pdf]

Supplementary Information

Two-Color Timestamping of Gene Expression with a Chemigenetic Reporter System

Henriette Lämmermann,[1] Jade Nguyen,[2] Juan F. Tamez-Fernández,[1] Fabien Kuttler,[3] Julien Bortoli Chapalay,[3] Marc Chambon,[3] Gerardo Turcatti,[3] Pablo Rivera-Fuentes[1*]

1. Department of Chemistry, University of Zurich, Zurich, Switzerland.
2. Institute of Chemical Sciences and Engineering, École Polytechnique Fédérale de Lausanne, Lausanne, Switzerland.
3. Biomolecular Screening Facility, École Polytechnique Fédérale de Lausanne, Lausanne, Switzerland.

*Correspondence to: pablo.riverafuentes@uzh.ch

Table of Contents

1 SUPPLEMENTARY FIGURES AND TABLES 2
2 GENERAL INFORMATION 11
3 MAMMALIAN CELL CULTURE 12
4 CLONING 12
4 ESTABLISHMENT OF THE STABLE CELL LINE 17
6 VALIDATION EXPERIMENTS 17
7 FLUORESCENCE MICROSCOPY 20
8 IMAGE ANALYSIS 21
9 FLOW CYTOMETRY 21
10 HIGH-CONTENT SCREENING 22
11 SYNTHESIS 24
12 SUPPLEMENTARY REFERENCES 38

1 Supplementary Figures and Tables

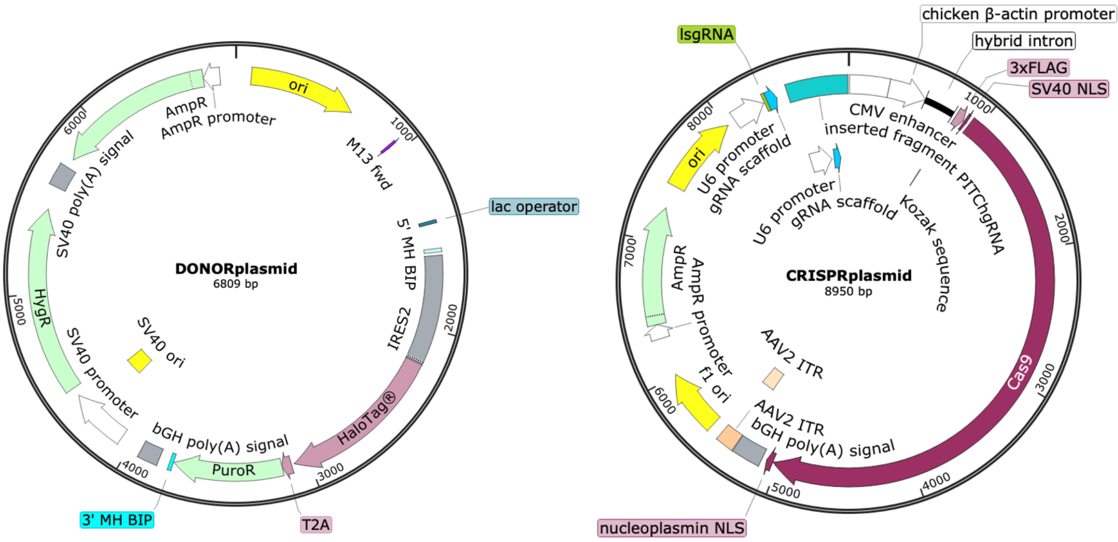

Supplementary Figure 1. Plasmid maps of DONOR and CRISPR plasmids. Created with SnapGene 7.1.1.

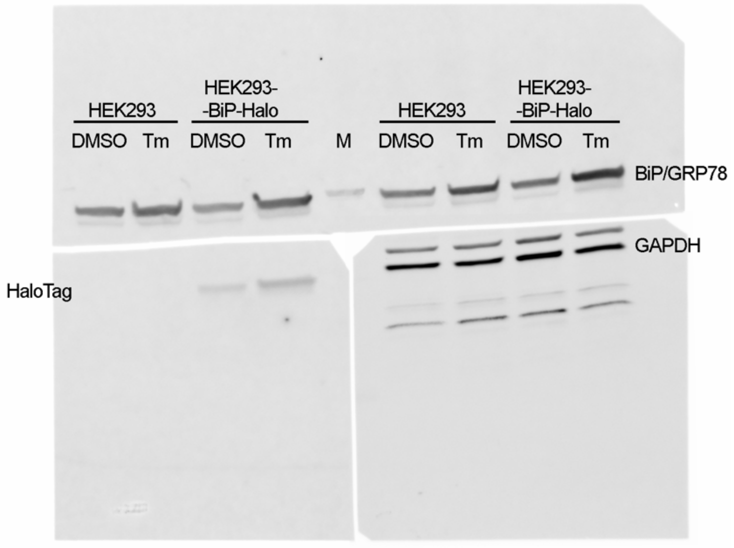

Supplementary Figure 2. Full-scale image of Western blot (stitched image). HEK293 or HEK293-BiP-HT cells were incubated with DMSO (0.1%) or tunicamycin (Tm, 5 µg mL^{-1}) for 24 h. Per lane, 35 µg total protein were loaded. Anti-BiP/GRP78 and anti-HaloTag antibodies were visualized with an Alexa Fluor Plus 488 nm conjugated secondary antibody, anti-GAPDH was visualized with an Alexa Fluor Plus 647 nm conjugated antibody.

Supplementary Table 1. DNA sequences downstream of *HSPA5* (BiP/GRP78) before and after genome editing. GREEN: *HSPA5*, *ORANGE* and *PURPLE*: microhomology sequences, blue: IRES-*HaloTag*-T2A-*PuroR* insert, **RED**: deletions.

DNA sequence of HEK293 cells around genome editing site before editing:

…AGCAAACTCTATGGAAGTGCAGGCCCTCCCCCAACTGGTGAAGAGGATACAGCAGAAAAAG
ATGAGTTGTAGACACTGATCTGCTAGTGCTGTAATATTGTAAATACTGGACTCAGGAACTTTTGT
TAGGAAAAAATTGAAAGAACTTAAGTCTCGAATGTAATTGGAATCTTCACCTCAGAGTGG*AGTTG*
*AAACTGCTATAGCCTAAGCGGCTGTTTACTGCTTT*TCATTAGCAGTTGCTCACATGTCTTTGGGT
GGGGGGGAGAAGAAGAATTGGCCATCTTAAAAAGCGGGTAAAAAACCTGGGTTAGGGTG…

DNA sequence of allele 1 in HEK293-BiP-HT cells around genome editing site after editing:

…AGCAAACTCTATGGAAGTGCAGGCCCTCCCCCAACTGGTGAAGAGGATACAGCAGAAAAAG
ATGAGTTGTAGACACTGATCTGCTAGTGCTGTAATATTGTAAATACTGGACTCAGGAACTTTTGT
TAGGAAAAAATTGAAAGAACTTAAGTCTCGAATGTAATTGGAATCTTCACCTCAGAGTGG*AGTTG*
*AAACTGCTATAGCCT*tatcgaattggatccgcccctctccctcccccccc**C**taacgttactggccgaagccgcttggaataagg
ccggtgtgcgtttgtctatatgttattttccaccatattgccgtcttttggcaatgtgagggcccggaaacctggccctgtcttcttgacgagcattc
ctaggggtctttcccctctcgccaaaggaatgcaaggtctgttgaatgtcgtgaaggaagcagttcctctggaagcttcttgaagacaaaca
acgtctgtagcgaccctttgcaggcagcggaaccccccacctggcgacaggtgcctctgcggccaaaagccacgtgtataagatacacc
tgcaaaggcggcacaaccccagtgccacgttgtgagttggatagttgtggaaagagtcaaatggctctcctcaagcgtattcaacaaggg
gctgaaggatgcccagaaggtaccccattgtatgggatctgatctggggcctcggtacacatgctttacatgtgtttagtcgaggttaaaaaa
acgtctaggccccccgaaccacggggacgtggttttcctttgaaaaacacgatgataatatggccacaacc*atggcagaaatcggtactg*
gctttccattcgacccccattatgtggaagtcctgggcgagcgcatgcactacgtcgatgttggtccgcgcgatggcacccctgtgctgttcct
gcacggtaacccgacctcctcctacgtgtggcgcaacatcatcccgcatgttgcaccgacccatcgctgcattgctccagacctgatcggta
tgggcaaatccgacaaaccagacctgggttatttcttcgacgaccacgtccgcttcatggatgccttcatcgaagccctgggtctggaagag
gtcgtcctggtcattcacgactggggctccgctctgggtttccactgggccaagcgcaatccagagcgcgtcaaaggtattgcatttatggag
ttcatccgccctatcccgacctgggacgaatggccagaatttgcccgcgagaccttccaggccttccgcaccaccgacgtcggccgcaag
ctgatcatcgatcagaacgtttttatcgagggtacgctgccgatgggtgtcgtccgcccgctgactgaagtcgagatggaccattaccgcga
gccgttcctgaatcctgttgaccgcgagccactgtggcgcttcccaaacgagctgccaatcgccggtgagccagcgaacatcgtcgcgct
ggtcgaagaatacatggactggctgcaccagtcccctgtcccgaagctgctgttctggggcaccccaggcgttctgatcccaccggccga
agccgctcgcctggccaaaagcctgcctaactgcaaggctgtggacatcggcccgggtctgaatctgctgcaagaagacaacccggac
*ctgatcggcagcgagatcgcgcgctggctgtccacgctcgagatttccggc*tacaagggaagcggagagggcagaggaagtctgctaa
catgcggtgacgtcgaggagaatcctggacct*atgaccgagtacaagcccacggtgcgcctcgccacccgcgacgacgtcccccggg*
ccgtacgcaccctcgccgccgcgttcgccgactaccccgccacgcgccacaccgtcgacccggaccgccacatcgagcgggtcaccg
agctgcaagaactcttcctcacgcgcgtcgggctcgacatcggcaaggtgtgggtcgcggacgacggcgccgcggtggcggtctggac
cacgccggagagcgtcgaagcgggggcggtgttcgccgagatcggcccgcgcatggccgagttgagcggttcccggctggccgcgca
gcaacagatggaaggcctcctggcgccgcaccggcccaaggagcccgcgtggttcctggccaccgtcggcgtctcgcccgaccacca
gggcaagggtctgggcagcgccgtcgtgctccccggagtggaggcggccgagcgcgccggggtgcccgccttcctggagacctccgc
gccccgcaacctccccttctacgagcggctcggcttcaccgtcaccgccgacgtcgaggtgcccgaaggaccgcgcacctggtgcatga

*cccgcaagcccggtgcctgaAAGCGGCTGTTTACTGCTTT*TCATTAGCAGTTGCTCACATGTCTTTGGGT
GGGGGGGAGAAGAAGAATTGGCCATCTTAAAAAGCGGGTAAAAAACCTGGGTTAGGGTG…

*red highlight: sequencing revealed the deletion of one C at the start of the IRES sequence. This deletion was introduced during the cloning of the DONOR plasmid and mistakenly overlooked. Nevertheless, all validation experiments prove that the IRES is functional.

DNA sequence of allele 2 in HEK293-BiP-HT cells around genome editing site after editing:

…AGCAAACTCTATGGAAGTGCAGGCCCTCCCCCAACTGGTGAAGAGGATACAGCAGAAAAAG
ATGAGTTGTAGACACTGATCTGCTAGTGCTGTAATATTGTAAATACTGGACTCAGGAACTTTTGT
TAGGAAAAAATTGAAAGAACTTAAGTCTCGAATGTAATTGGAATCTTCACCTCAGAGTGG*AGTTG
AAACTGCTA***TAGCCTA***AGCGGCTGTTTACTGCTTT*TCATTAGCAGTTGCTCACATGTCTTTGGGT
GGGGGGGAGAAGAAGAATTGGCCATCTTAAAAAGCGGGTAAAAAACCTGGGTTAGGGTG…

Supplementary Table 2. Overview of mutations found in whole-genome sequencing in HEK293-BiP-HT cells compared to HEK293 cells. Of the 2335 mutations, only 33 are homozygous, and none of these 33 mutations are in a coding region.

**Total mutations (2335)**	**In coding region (26/2335)**	**Mutation type**	**Impact**
Insertions (49)	Insertions (1/49)	In-frame insertion (1/1)	Moderate
Deletions (60)	Deletions (0/60)	–	–
SNPs (2226)	SNPs (25/2226)	Synonymous (9/25)<br>Missense (15/25)<br>Nonsense (1/25)	Low<br>Moderate<br>High

Supplementary Table 3. List of genes highly or moderately impacted by mutations.

**Gene**	**Protein**	**Function**	**Mutation**
VPS41	Vacuolar protein sorting-associated protein 41 homolog	regulation of transport of newly synthesized lysosomal membrane and secretory proteins	Nonsense
IFFO1	Non-homologous end joining factor IFFO1	involved in immobilization of broken DNA ends and suppression of chromosome translocation during DSBs	In-frame insertion of two amino acids

CASZ1	Zinc finger protein castor homolog 1	transcriptional activator	Single base missense mutation
BROX	BRO1 domain-containing protein BROX	involved in the ESCRT (endosomal sorting complex required for transport) pathway	Single base missense mutation
OR1C1	Olfactory receptor 1C1	odorant receptor	Single base missense mutation
SLC4A7	Sodium bicarbonate cotransporter 3	regulates intracellular pH and may play a role in bicarbonate salvage in secretory epithelia	Single base missense mutation
TIGD2	Tigger transposable element-derived protein 2	DNA transposon, plays a role in evolution	Single base missense mutation
GRPEL2	GrpE protein homolog 2, mitochondrial	helps translocation of transit peptide-containing proteins into the mitochondrial matrix in an ATP-dependent manner	Single base missense mutation
TRBV6-3	T cell receptor beta variable 6-3	participates in apha-beta T cell receptors' recognition (essential to the immune response)	Single base missense mutation
TAF1L	Transcription initiation factor TFIID subunit 1-like	may act as a functional substitute for TAF1/TAFII250 during male meiosis	Single base missense mutation
ATF7	Cyclic AMP-dependent transcription factor ATF-7	stress-responsive chromatin regulator that plays a role in innate immunological memory and adipocyte differentiation	Single base missense mutation
MEDAG	Mesenteric estrogen-dependent adipogenesis protein	involved in adipocyte differentiation, lipid accumulation, and glucose uptake in mature adipocytes	Single base missense mutation
ZNF781	Zinc finger protein 781	may be involved in transcriptional regulation	Single base missense mutation
SSC5D	Soluble scavenger receptor cysteine-rich	binds to extracellular matrix proteins	Single base missense mutation

	domain-containing protein SSC5D		
USP29	Ubiquitin carboxyl-terminal hydrolase 29	thiol-dependent hydrolysis of ester, thioester, amide, and peptide bonds formed by the C-terminal Gly of ubiquitin	Single base missense mutation
PCK1	Phosphoenolpyruvate carboxykinase, cytosolic [GTP]	regulates processes that control levels of intermediates in the citric acid cycle	Single base missense mutation

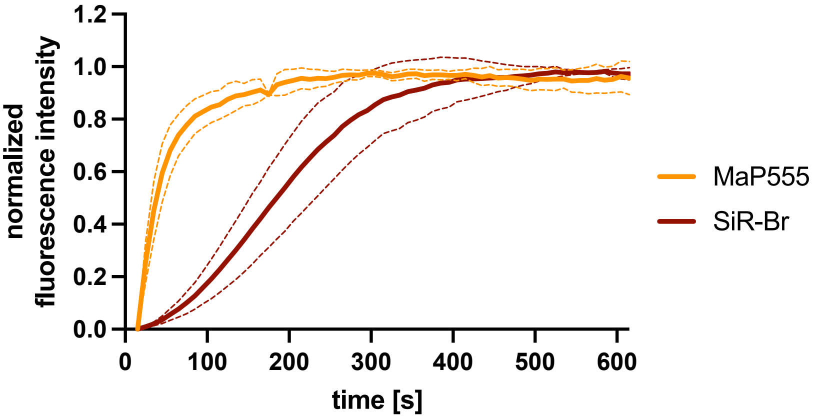

Supplementary Figure 3. Dye kinetics. HEK293-BiP-HT cells were incubated with MaP555 (100 nM) or SiR-Br (100 nM). Images (561 and 638 nm) were acquired every 10 s starting 15 s after addition. For each dye, the intensity values from three different fields of view were plotted. The solid line represents the mean, the dashed lines represent the standard deviation.

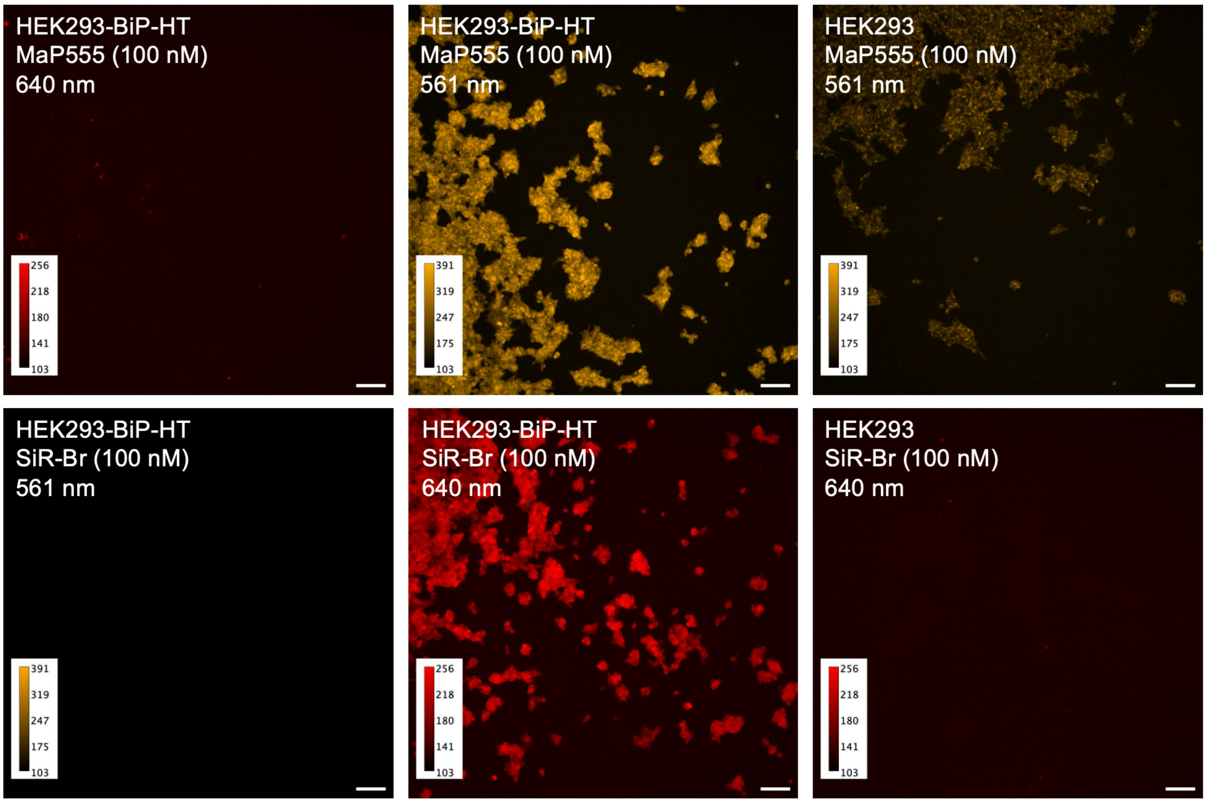

Supplementary Figure 4. Background fluorescence of MaP555 and SiR-Br. HEK293 or HEK293-BiP-HT cells were incubated with MaP555 (100 nM) or SiR-Br (100 nM) for 30 min (no wash), then images were captured (561 nm (MaP555) or 640 nm (SiR-Br) laser). Scale bar: 100 µm.

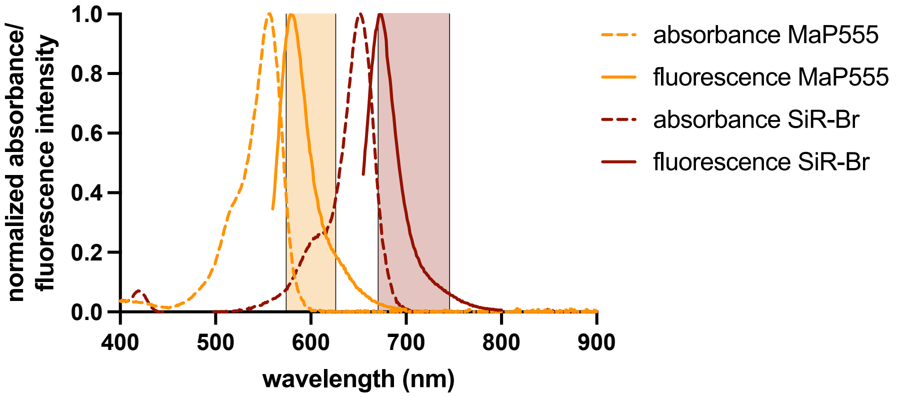

Supplementary Figure 5. Normalized absorbance and fluorescence spectra of MaP555 and SiR-Br (5 µM in 1x PBS with 0.1% SDS). Dashed lines: absorbance, solid lines: fluorescence (excitation at 555 or 650 nm). Colored regions indicate transmission band of microscopy filters (orange: 600/52, red: 708/75).

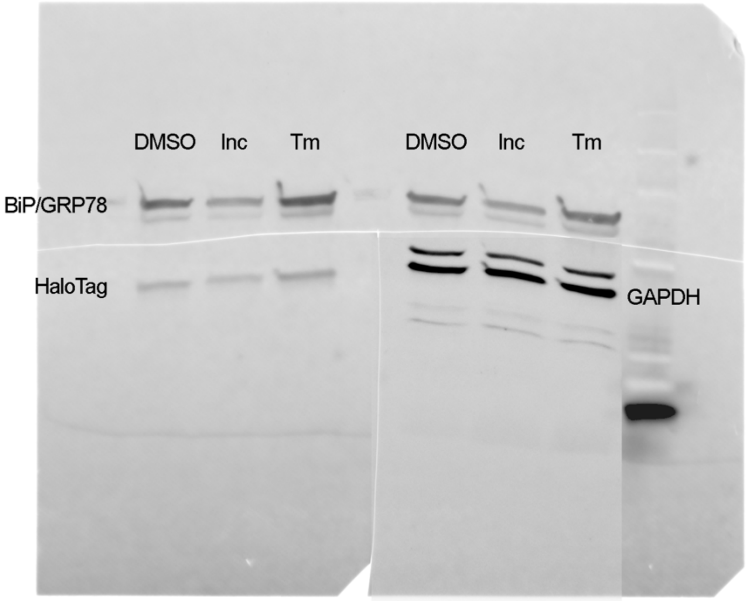

Supplementary Figure 6. Western blot (stitched image) to verify whether the labeling procedure induces BiP/GRP78 or HaloTag upregulation. HEK293-BiP-HT cells were incubated with DMSO (0.1%) or tunicamycin (Tm, 5 µg mL^{-1}) for 24 h or subjected to the labeling procedure (Inc; MaP555 (100 nM) for 30 min, wash for 30 min, followed by SiR-Br (100 nM) and DMSO (0.1%) for 24 h). Per lane, 35 µg total protein were loaded. Anti-BiP/GRP78 and anti-HaloTag antibodies were visualized with an Alexa Fluor Plus 488 nm conjugated secondary antibody, anti-GAPDH was visualized with an Alexa Fluor Plus 647 nm conjugated antibody.

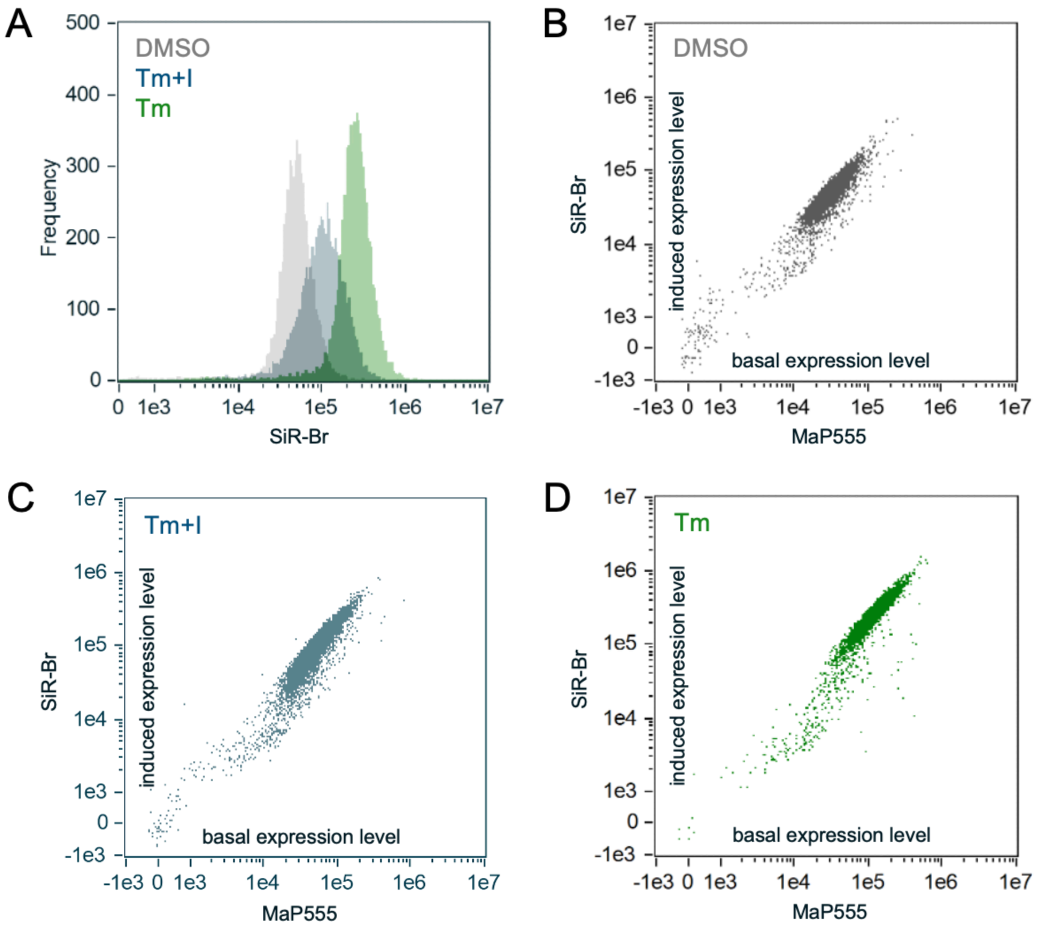

Supplementary Figure 7. Flow cytometry experiment. HEK293-BiP-HT cells were incubated with MaP555 (100 nM, 30 min), washed, and then incubated with three different treatments: DMSO (0.1%), Tm (2 µg mL^{-1}), or Tm (2 µg mL^{-1}) and PF-429242 (**I**, 10 µM), together with SiR-Br (100 nM). **A**, Comparison of the three treatments. Gates were set for single and live cells, histograms represent >13’000 cells per condition. **B to D,** Correlation of basal and induced expression levels of cells treated with DMSO (**B**), Tm+I (**C**), or Tm (**D**). Gates were set for single and live cells; scatter plots represent >13’000 cells.

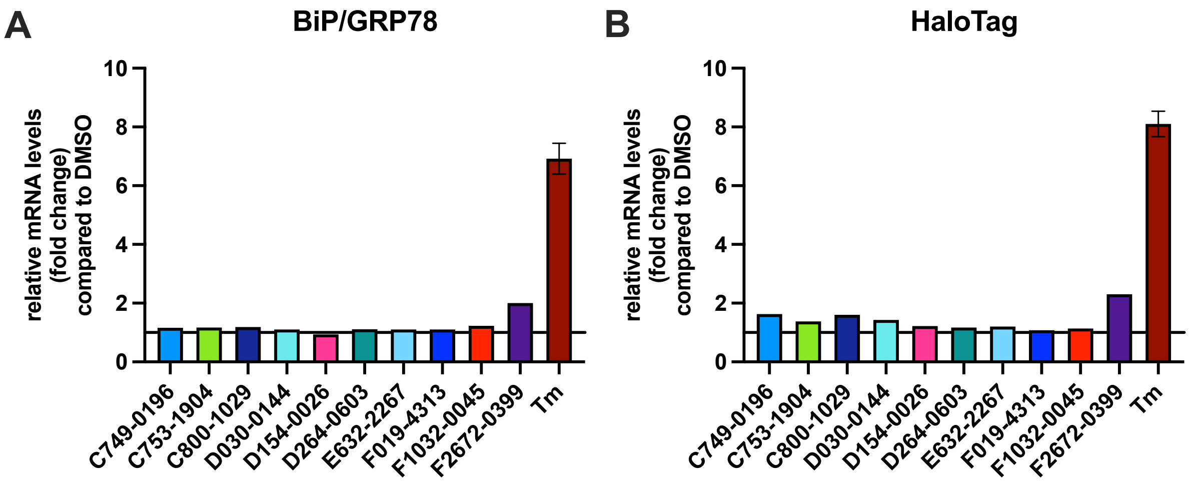

Supplementary Figure 8. RT-qPCR for hit validation. mRNA was isolated from HEK293-BiP-HT incubated with the 10 hits (10 µM), DMSO (0.1%), or tunicamycin (Tm, 2 mg mL^{-1}) for 24 h, and quantified by RT-qPCR. Shown is the relative abundance of **A**, BiP/GRP78 mRNA, and **B**, HaloTag mRNA compared to the DMSO control. For each condition, three replicates of the same sample were measured.

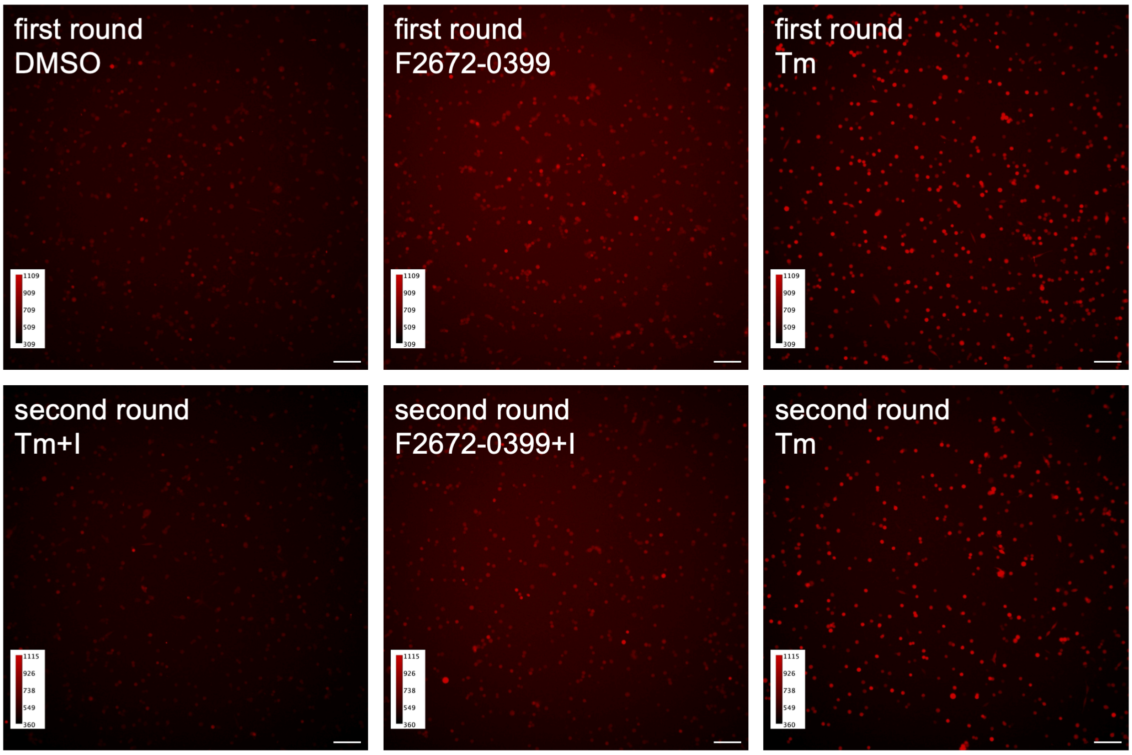

Supplementary Figure 9. Screening images from first and second rounds. HEK239-BiP-HT cells were incubated with MaP555 (100 nM, 30 min), washed, then incubated with DMSO (0.1%), F2672-0399 (10 µM), or Tm (2 µg mL^{-1}) and SiR-Br (100 nM) for 24 h. In the second round, the compounds were screened in the presence of the inhibitor PF429242 (I, 10 µM). Shown are representative images for each condition (SiR-Br channel). Scale bar: 100 µm.

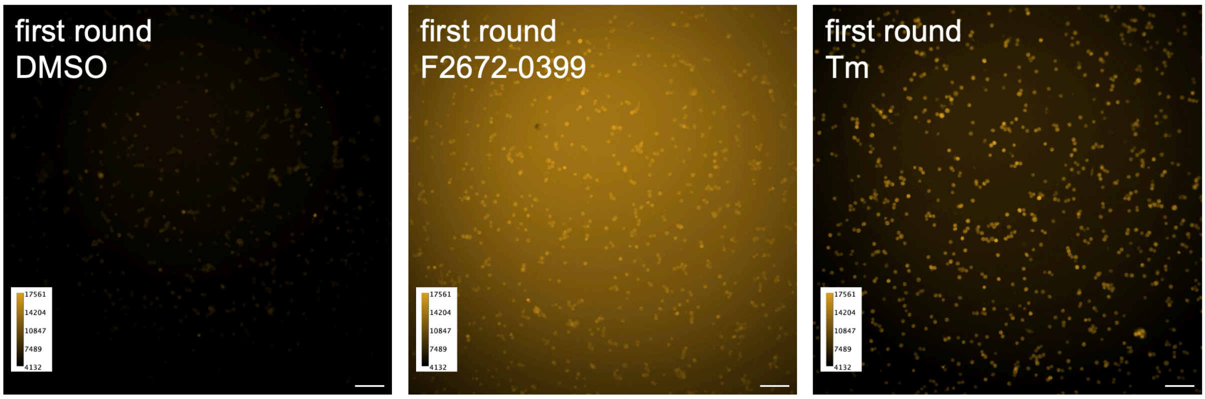

Supplementary Figure 10. Screening images from one-color screening. HEK239-BiP-HT cells were incubated with MaP555 (100 nM, 30 min), washed, then incubated with DMSO (0.1%), F2672-0399 (10 µM), or Tm (2 µg mL^{-1}) and MaP555 (100 nM) for 24 h. Shown are representative images for each condition (MaP555 channel). Scale bar: 100 µm.

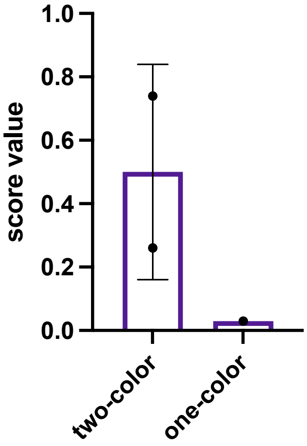

Supplementary Figure 11. Comparison of score values of compound F2672-0399. Plotted are the values obtained from the screening without inhibitor using either the two- or the one-color labeling strategy. Dots represent data points, bars represent the mean, and error bars represent the standard deviation.

2 General information

All reagents were purchased from commercial sources and used as received. Anhydrous solvents were procured from Acros Organics and used as received. NMR spectra were acquired on Bruker 400, 600, or 800 instruments. ^{1}H NMR chemical shifts are reported in ppm relative to $SiMe_4$ (δ = 0) and were referenced internally with respect to residual protons in the solvent (δ = 7.26 for $CDCl_3$, δ = 3.31 for CD_3OD, and δ = 2.50 for $(CD_3)_2SO$). Coupling constants are reported in Hz. ^{13}C NMR chemical

shifts are reported in ppm relative to $SiMe_4$ (δ = 0) and were referenced internally with respect to solvent signal (δ = 77.16 for $CDCl_3$, δ = 49.00 for CD_3OD, and δ = 39.52 for $(CD_3)_2SO$). Preliminary peak assignments are based on calculated chemical shifts, multiplicity, and 2D experiments (COSY, HSQC, HMBC). Automatic flash column chromatography was carried out using prepacked Buchi Reveleris SiO_2 or C18 cartridges on a Buchi Reveleris PREP instrument. Liquid Chromatography-Mass Spectrometry (LC-MS) experiments were performed on a Shimadzu LCMS 2020 with an ESI Single Quadrupole Detector or an Agilent 1290 Infinity II LC System with an Agilent InfinityLab LC/MSD. High-resolution mass spectra (HRMS) were acquired on a Xevo G2-S QTof spectrometer (Waters) using electrospray ionization (ESI) by staff at the EPFL mass spectrometry service or on a Dionex Ultimate 3000 UHPLC system (Thermo Fisher) connected to a QExactive MS with a heated ESI source (Thermo Fisher) at UZH. IUPAC names of all compounds are provided and were determined using ChemDraw Professional 20.1.

3 Mammalian cell culture

HEK293 and HEK293-BiP-HT cells were grown in Dulbecco's Modified Eagle Medium (DMEM) supplemented with fetal bovine serum (FBS, 10%) and 1× antibiotic-antimycotic solution (penicillin, streptomycin, amphotericin B) at 37 °C in a 5% CO_2 environment. The cells were passaged at 80–90% confluence.

4 Cloning

Amplification of plasmid DNA. All plasmids were obtained from Addgene: 1. pCAG-Cre-IRES2-GFP #26646, 2. pX330A-1x2 #58766, 3. pX330S-2-PITCh #63670, 4. pX330A-FBL/PITCh #63671, 5. pCRIS-PITChv2-FBL #63672, 6. TUBB5-Halo #64691, 7. pcDNA3.1(+)-GRP78/BiP #32701. Lysogeny broth (LB) medium and LB agar plates were prepared with either carbenicillin (100 µg mL^{-1}), kanamycin (50 µg mL^{-1}), or spectinomycin (50 µg mL^{-1}). Amplification of plasmid DNA was accomplished by streaking from a bacterial stab onto an LB agar plate. For plasmids pCAG-Cre-IRES2-GFP and TUBB5-Halo, liquid cultures (5 mL) were inoculated and incubated over night (37 °C, 300 rpm), then plasmid DNA was purified using the

QIAprep Spin Miniprep Kit (Qiagen) according to the manufacturer's instructions. For the other plasmids, liquid starter cultures (3 mL) were inoculated and incubated (37 °C, 8 h, 300 rpm), then liquid cultures (100 mL) were inoculated with these starter cultures (200 µL) and incubated over night (37 °C, 100 rpm). Plasmid DNA was purified using the QIAGEN Plasmid Midi Kit following the manufacturer's instructions.

For amplification of plasmid DNA after transformation into *E. coli*, liquid starter cultures (3 mL) were inoculated and incubated (37 °C, 8 h, 300 rpm). These starter cultures were used to inoculate liquid cultures (100 µL for 50 mL). After overnight incubation (37 °C, 100 rpm), the plasmid DNA was purified using either the QIAGEN Plasmid Midi Kit or the QIAGEN Plasmid Plus Midi Kit according to the manufacturer's instructions.

General procedure for Gibson assembly. For Gibson assembly, the fragments were first amplified by PCR using primers that were generated using SnapGene (minimum 15 bp overlap) and further optimized to improve melting temperatures and GC content, as well as to minimize hairpin formation, dimerization, and repeating motifs. All primers were supplied by Microsynth AG and are listed below. For PCR, Phusion High-Fidelity PCR Master Mix with HF Buffer (NEB) was used. For each fragment, 5 × 25 µL reactions with 0.1, 0.5, 1, 2, and 5 ng template DNA per reaction were carried out, following the instructions provided by NEB. After DpnI-digestion (NEB, 10 units per PCR reaction), the PCR reactions were analyzed by agarose gel electrophoresis and purified either by using the QIAquick PCR Purification Kit (Qiagen) or by gel extraction using the QIAquick Gel Extraction Kit (Qiagen). The Gibson assembly reactions (20 µL) were carried out using the 2× Gibson Assembly Master Mix (NEB). Assembly reactions were incubated at 50 °C for 15 min, then 5 µL were used to transform NEB 5-α competent *E. coli*, following the manufacturer's protocol. Purification of plasmid DNA was achieved using the QIAGEN Plasmid Midi Kit.

CRIS-PITCh method.[1] In this method, transfection with two plasmids is used to incorporate the desired sequence into the genome at the desired location. The method makes use of the microhomology-mediated end joining (MMEJ) DNA double-strand break repair mechanism. The first (DONOR) plasmid contains the insert sequence flanked by short microhomology regions (5-25 bp) that are complementary to the flanking regions of the DNA cut site. The second (CRISPR) plasmid contains the Cas9 enzyme sequence together with two guide RNAs. One guide RNA directs Cas9 to cut the DONOR plasmid to linearize the insert flanked by microhomologies, the other guide RNA directs Cas9 to cut the genome at the desired location.

4.1 DONOR plasmid containing the insert sequence

First, the IRES-HaloTag-T2A-PuroR sequence was assembled, then the microhomologies complementary to the flanking regions of the cut site in the 3'-untranslated region downstream of BiP/GRP78 were introduced.

Three-fragment Gibson assembly. For the three-fragment Gibson assembly, the IRES2-fragment was obtained from plasmid pCAG-Cre-IRES2-GFP, the HaloTag-fragment was amplified from the plasmid TUBB5-Halo, and the plasmid pCRIS-PITChv2-FBL was used for the backbone fragment. The PCR reactions of the IRES2- and HaloTag-fragments were purified by PCR purification, whereas the backbone fragment was purified by gel extraction. The Gibson assembly was performed with a 3:3:1 ratio of IRES2-fragment, HaloTag-fragment, and backbone (22.8 fmol of each fragment, 7.60 fmol of backbone).

Primers for three-fragment Gibson assembly:

- IRES2 fragment:
  IRES2.fwd: 5'-TCTCACAGGAGCTTGATATCGAATTGGGATCCG-3'
  IRES2.rev: 5'-CCGATTTCTGCCATGGTTGTGGCCATATTATCATC-3'
- HaloTag fragment:
  Halo.fwd: 5'-CCATGGCAGAAATCGGTACTGGCTTTCCATTCG-3'
  Halo.rev: 5'-CCCTTGTAGCCGGAAATCTCGAGCGT-3'
- Backbone fragment:
  vector.fwd: 5'-TTCCGGCTACAAGGGAAGCGGAGAG-3'
  vector.rev: 5'-ATATCAAGCTCCTGTGAGAGGAACCAAACACG-3'

Gibson assembly for microhomology exchange. For the second Gibson assembly, both fragments were amplified from the plasmid obtained in the first Gibson assembly. The "insert" fragment (IRES2-HaloTag-T2A-PuroR) was amplified by two-step PCR because the melting temperature of the primers containing the microhomologies exceeded 72 °C. Purification of the "insert" fragment was accomplished by PCR purification. The amplified "backbone" fragment was purified by gel extraction. Gibson assembly was performed with a 3:1 ratio of "insert" to "backbone" (26.5 fmol of "insert", 8.84 fmol of "backbone").

Primers for second Gibson assembly:

- "insert":
  ins.fwd: 5'-GCGTTACATAGCATCGTACGCGTACGTGTTTGGAGTTGAAACTGCTATAGCCTTATCGAATTGGGATC-3'

ins.rev: 5'-CTAGAGCATCGTACGCGTACGTGTTTGGAAAGCAGTAAACAGCCGCTTTCAGGCACCGGGCTT-3'

(microhomology sequences underlined)

- “backbone”:

  vect.fwd: 5'-CGTACGATGCTCTAGAATGC-3'

  vect.rev: 5'-CGATGCTATGTAACGCGGAA-3'

4.2 CRISPR plasmid containing Cas9 and guide RNAs

First, the locus-specific gRNA (ls-gRNA) sequence was cloned into the plasmid pX330A-1x2, then the PITCh-gRNA sequence together with its U6 promoter was inserted by Golden Gate cloning.

ls-gRNA oligo design. The online tool “GPP sgRNA Designer” (CRISPRko) by the Broad Institute was used to search for the most suitable ls-gRNA. A sequence of 170 or 250 bp of the 3'-UTR immediately downstream of the last exon of BiP was used as the search template. From the top five picks of each search, the ls-gRNA that was first in one and second in the other search was chosen. The two oligonucleotides (top and bottom strand) consisting of the ls-gRNA sequence and the overhangs matching those generated by the restriction enzyme BpiI were ordered from Microsynth AG.

ls-gRNA oligo1: 5'-caccgTGAAACTGCTATAGCCTAAG-3'

ls-gRNA oligo2: 5'-aaacCTTAGGCTATAGCAGTTTCAc-3'

Phosphorylation and annealing of oligos. For the cloning of the ls-gRNA into the plasmid pX330A-1x2, the oligonucleotides were first phosphorylated and annealed to each other. The following reaction mixture was set up:

1 µL	oligo1 (100 µM)
1 µL	oligo2 (100 µM)
1 µL	10× T4 DNA Ligase Reaction Buffer (NEB)
0.5 µL	T4 PNK (NEB)
6.5 µL	ddH_2O

The mixture was incubated at 37 °C for 30 min, then at 65 °C for 20 min, then at 95 °C for 5 min, then the temperature was ramped down to 25 °C at 0.1 °C s^{-1}. Finally, the mixture was diluted 1:50 in ddH_2O.

Digestion and dephosphorylation of plasmid. The plasmid pX330A-1x2 was digested and dephosphorylated in one reaction:

5 µg	plasmid DNA
5 µL	10× FastDigest Buffer (Thermo Fisher)
5 µL	FastDigest BpiI (Thermo Fisher)
5 µL	FastAP Thermosensitive Alkaline Phosphatase (Thermo Fisher)
0.5 µL	DL-dithiothreitol (DTT, 100 mM)
to 50 µL ddH$_2$O	

This mixture was incubated at 37 °C for 1 h.

Ligation. The ligation reaction was performed twice, once with the oligo duplex and once without, as a negative control. The following mixture was assembled:

50 ng	digested plasmid
1 µL	diluted oligo duplex
10 µL	2× Quick Ligase Reaction Buffer (NEB)
1 µL	Quick Ligase (NEB)
to 20 µL ddH$_2$O	

The reaction was incubated at room temperature for 30 min.

Transformation and plasmid DNA isolation. Transformation of One Shot Stbl3 chemically competent E. coli (Thermo Fisher) was carried out using 5 µL of the reaction mixture and following the manufacturer's instructions. For purification of plasmid DNA, the QIAGEN Plasmid Plus Midi Kit was used.

Golden Gate cloning. For Golden Gate cloning, the NEB Golden Gate Assembly Kit (BsaI-HFv2) was used. The reaction was carried out with the plasmid obtained from the previous cloning step and the plasmid pX330S-2-PITCh, following the protocol provided by NEB. After incubation (5 min at 37 °C, then 5 min at 60 °C), the reaction mixture (5 µL) was used to transform XL1-Blue supercompetent cells (Agilent) according to the manufacturer's instructions. The LB agar plates for blue-white screening (LB medium with 80 µg mL^{-1} X-gal and 20 mM IPTG) were also prepared according to this protocol. The QIAGEN Plasmid Plus Midi Kit was used for plasmid DNA purification.

4 Establishment of the stable cell line

Co-transfection and puromycin selection. For transfection of HEK293 cells, jetPRIME reagent (Polyplus) was employed according to the manufacturer's instructions. A 1:2 ratio of the CRISPR to DONOR plasmid was used (0.6 µg total plasmid DNA per 100'000 cells). HEK293 cells were seeded into a 6-well plate (2 mL per well) with the following seeding densities: 50’000, 100’000, and 200’000 cells per well. After overnight incubation, the cells were transfected and incubated for 72 h. A control with non-transfected cells was maintained for each cell concentration. For selection, growth medium containing 0.7 µg mL^{-1} puromycin was used. The cells were incubated with selection medium for a week. Every day, the medium was removed, the cells were washed twice with PBS, and fresh selection medium was added. After 6 to 7 days, single colonies were picked using a curved glass tip to scrape the cells from the surface and a pipette to transfer the colonies to wells of a 24-well plate (0.5 mL per well). For the first few days, the cells were cultured in growth medium containing 0.35 µg mL^{-1} puromycin, then in standard growth medium.

Fluorescence-activated cell sorting. After puromycin cell selection, the colonies were grown for several days and then incubated with MaP555 for 30 min, washed twice with PBS, trypsinized, spun down, washed with PBS, and resuspended in 300 µL PBS. Propidium iodide (PI) solution (0.1 µL, 1 mg mL^{-1}) was added to the sample as a cell viability marker. The samples were measured, and the single cells were sorted into 96-well plates (100 µL per well) by the staff at the Flow Cytometry Core Facility (FCCF) at EPF Lausanne using a BD FACSAria Fusion flow cytometer.

6 Validation experiments

6.1 Western Blot

Protein lysates. Both HEK293 and HEK293-BiP-HT cells were incubated with tunicamycin (5 µg mL^{-1}) or DMSO (1 µL mL^{-1}) in growth medium for 24 h at 37 °C. Protein lysates were obtained using CytoBuster Protein Extraction Reagent following the manufacturer’s instructions. Protease Inhibitor Cocktail P8340 (Sigma) was additionally added during cell lysis (1 µL per 10^6 cells). The protein concentration of the lysates was determined with the BCA protein assay kit (Novagen).

Blotting. Samples of the lysates were loaded on precast 4–20% Mini-PROTEAN TGX Stain-Free Protein Gels (Bio-Rad, 10 well, 30 µL). For Western blotting, a Trans-Blot Turbo Transfer System (Bio-Rad) with Trans-Blot Turbo Mini 0.2 µm PVDF Transfer Packs (Bio-Rad) was used. For BiP, the high MW program was used. For HaloTag, the low MW program was used. After transfer, the membranes were left to dry at room temperature.

Antibody incubation. The membranes were reactivated with MeOH (30 s), then rinsed with water (5 min) and incubated with blocking solution (5% skim milk in PBST (PBS containing 0.05% Tween20)) for 2 h at room temperature. After rinsing with PBST (5 min), the membranes were incubated with the primary antibody in PBST with 1% skim milk at 4 °C over night. For BiP, GRP78 monoclonal antibody (1H11-1H7) (Thermo Fisher, 1 µg mL^{-1}), for HaloTag, Anti-HaloTag Monoclonal Antibody (Promega, 1 µg mL^{-1}), and for GAPDH, GAPDH polyclonal antibody (Invitrogen, 1 µg mL^{-1}) were used. The membranes were rinsed with PBST (3 x 10 min), then incubated with a solution of secondary antibodies (Goat anti-Mouse IgG (H+L) Highly Cross-Adsorbed Secondary Antibody, Alexa Fluor Plus 488 (Thermo Fisher) and Donkey anti-Rabbit IgG (H+L) Cross-Adsorbed Secondary Antibody, Alexa Fluor 647 (Thermo Fisher), both 0.4 µg mL^{-1}) in PBST with 1% skim milk. After incubation, the membranes were rinsed in PBST (4 x 5 min), followed by PBS (2 x 5 min). Imaging was performed with a Bio-Rad ChemiDoc MP Imaging System.

6.2 RT-qPCR

RNA isolation. Both HEK293 and HEK293-BiP-HT cells were incubated with tunicamycin (2 µg mL^{-1}) or DMSO (1 µL mL^{-1}) in growth medium for 6 h at 37 °C. Total RNA was isolated using the PureLink RNA Mini Kit (Invitrogen) with TRIzol reagent (Invitrogen) according to the manufacturer's instructions. RNA yield and quality were examined by absorbance measurements (Multiskan Sky Microplate Spectrophotometer –Thermo Fisher); 260 nm, ratios 260/80 and 260/30) and agarose gel electrophoresis.

First-strand DNA synthesis. The cDNA synthesis was performed using SuperScript III Reverse Transcriptase (Invitrogen) according to the manufacturer's protocol. Oligo$(dT)_{20}$ primer was used for the amplification.

General PCR procedure. In general, 10 µL PCR reactions were set up in FrameStar 384-well skirted PCR plates (4titude, clear wells, clear frame) using iQ SYBR Green

Supermix (Bio-Rad) and primer concentrations of 0.3 µM. A QuantStudio 7 Pro Real-Time PCR System (Applied Biosystems) was employed, the run program is reported below.

PCR program:

- 50 °C, 2 min
- 95 °C, 10 min
- 40 cycles: 95 °C, 15 s
  60 °C, 1 min
- melt curve: 95 °C, 15 s
  60 °C, 1 min
  ramp up to 95 °C with 0.05 °C s^{-1}

Primer efficiency test. For each target (HaloTag, BiP, GAPDH), three sets of primers were tested. For each primer set, reactions were performed with five different DNA concentrations (100, 20, 4, 0.8, 0.16 ng) to generate a standard curve. Three replicates were done for each primer set. By plotting the Cq value against $\log_{10}$([cDNA]), the primer efficiency was calculated from the slope of the linear fit with the following equation (1):

$$E = 10^{-1/slope} - 1 \qquad (1)$$

Based on the calculated efficiencies and the R^2 values of the linear fits, the best primer sets were selected (see below). Additionally, melt curve analysis and agarose gel electrophoresis was used to evaluate the PCR reactions.

Primers for qPCR:

- HaloTag:
  HaloTag.fwd 5’-GAGTTCATCCGCCCTATCCC-3’
  HaloTag.rev 5’-ATCTCGACTTCAGTCAGCGG-3’
- BiP:
  BiP.fwd 5’-ACCCAGATGAAGCTGTAGCG-3’
  BiP.rev 5’-AGTTTGGTCATGACACCTCCC-3’
- GAPDH:
  GAPDH.fwd 5’-TCTGACTTCAACAGCGACACCC-3’
  GAPDH.rev 5’-TTCCTCTTGTGCTCTTGCTGGG-3’

qPCR. PCR reactions were carried out with 15 ng DNA per reaction. For each sample, three technical and three biological replicates were performed. The relative quantification ratio was calculated according to the Pfaffl method[2] using the following equation (2):

$$ratio = \frac{(E_{target})^{\Delta Cq_{target}\,(control-sample)}}{(E_{reference})^{\Delta Cq_{reference}\,(control-sample)}} \qquad (2)$$

6.3 Whole genome sequencing

DNA isolation. Genomic DNA (gDNA) samples of HEK293 and HEK293-BiP-HT cells were obtained using the DNeasy Blood & Tissue Kit (Qiagen) according to the manufacturer's instructions. Proteinase K and RNAse A were added to digest proteins and RNA, thus avoiding contamination for downstream applications. gDNA concentrations were obtained by Nanodrop measurements, and samples were handed to the Gene Expression Core Facility (GECF) at EPFL. The GECF staff confirmed gDNA concentrations with qubit measurements.

Library preparation. A TruSeq DNA PCR-Free library prep was performed starting from 1000 ng of genomic DNA, according to Illumina protocol 15036187 Rev. D. First, gDNAs were sheared with a Covaris S220. Tapestation TS4200 analysis was performed on gDNA before and after shearing.

Quality control. After library preparation, the libraries were quantified by qubit DNA HS, and profile analysis was performed by TapeStation TS4200. All libraries passed the quality control and had sufficient yields. Quantification was validated by qPCR using PowerUp SYBR green master mix (Thermo Fisher).

Sequencing. The libraries were sequenced using Illumina technology on a HiSeq 4000 with paired-end reads of 75 bases and 30× coverage (two lanes per sample, with >600 Mio reads per lane). The reads of the edited cells were trimmed for Truseq adapters with a minimal read length of 35 bp tolerated. The reads were aligned, and the data analysis was performed by the Bioinformatic Competence Center (BICC) at EPFL.

7 Fluorescence microscopy

Microscope. Imaging was performed using a spinning disk confocal microscope. A Nikon Eclipse Ti2-E microscope equipped with a CSU-W1 confocal scanner unit

(Yokogawa) and two sCMOS (Photometrics Prime 95B) cameras was employed. Light sources: diode laser 561 nm, and 638 nm or 640 nm. Objectives: oil-immersion 100× CFI Apo TIRF SR (NA = 1.49), oil-immersion 60× CFI Plan Apo (NA = 1.4), or 10× CFI Plan Apo λ. Emission filters: BrightLine single-band bandpass filters BP 525/50, BP 600/52, BP 708/75 nm. The microscope was operated using NIS-Element AR (Nikon) software.

Sample preparation. For microscopy experiments, 10’000–30’000 cells per well were seeded onto 8-well plates (Ibidi μ-slide 8-well, 300 μL) 1–3 days before imaging.

Initial validation experiment. HEK293-BiP-HT cells were incubated with DMSO (0.1%) or tunicamycin (1 μg mL^{-1}) for 16 h. The cells were washed with PBS, then imaging medium (FluoroBrite DMEM, Gibco) was added. The cells were incubated with MaP555 (100 nM) for 30 min in imaging medium, then imaged.

Kinetics experiment. The cells were washed with PBS, then imaging medium (FluoroBrite DMEM, Gibco) was added. Images were acquired every 10 s for 10 min immediately after addition of MaP555 (100 nM) or SiR-Br (100 nM).

Pulse-chase experiments. The cells were incubated with MaP555 (100 nM) in growth medium for 30 min, then the cells were incubated in growth medium (3 × 1 h). The cells were washed twice with 1× PBS, then imaging medium (FluoroBrite DMEM, Gibco) was added. Imaging medium containing SiR-Br (100 nM) and the treatment (DMSO (0.1%), Tm (2 μg mL^{-1}), or Tm (2 μg mL^{-1}) + PF-429242 (10 μM)) was added. Immediately after addition, images were acquired every 15 min over 15–20 h.

8 Image analysis

Image analysis was performed using a Python script. The code is provided on Zenodo (DOI: 10.5281/zenodo.15274965). Briefly, a mask was generated from the image of one fluorescence channel (561 nm), then a background subtraction was performed. The fluorescence intensity of the background-subtracted images was measured per field of view.

9 Flow cytometry

HEK293-BiP-HT cells were incubated with MaP555 (100 nM) in growth medium (30 min), then the cells were incubated in growth medium (3 × 30 min, then overnight). The cells were washed with 1× PBS, then SiR-Br (100 nM) and the treatment (DMSO

(0.1%), Tm (2 µg mL^{-1}), or Tm (2 µg mL^{-1}) + PF-429242 (10 µM)) was added, and the cells were incubated for 24 h. The cells were washed twice with 1× PBS, trypsinized, resuspended in growth medium, and pelleted by centrifugation (500 × g, 8 min, 4 °C). After resuspension in cold 1× PBS, the cells were incubated with LIVE/DEAD Fixable Violet Dead Cell Stain Kit (405 nm, Thermo Fisher, 1 µL mL^{-1}) at 4 °C for 30 min. The cells were pelleted by centrifugation (500 × g, 8 min, 4 °C) and resuspended in 1× PBS to a concentration of ~10^{7} cells mL^{-1}. Flow cytometry was performed using an Image Stream X analyzer at the Cytometry Facility at UZH. The following settings were used: Ch1 brightfield, Ch3 561 nm (577/35, 100 mW), Ch6 SSC (2 mW), Ch7 405 nm (457/45, 170 mW), Ch11 642 nm (702/85, 150 mW). Cells were gated for single cells, and at least 20'000 cells per condition were recorded. Analysis was performed with IDEAS image analysis software.

10 High-content screening

The high-content screening experiments were performed at the Biomolecular Screening Facility (BSF) at EPFL.

Cell culture. HEK293-BiP-HT cells were grown in Dulbecco's Modified Eagle Medium (DMEM) supplemented with fetal bovine serum (FBS, 10%) at 37 °C in a 5% CO_2 environment. The cells were passaged at 80–90% confluence.

Plate Preparation. All screened compounds, if used the inhibitor PF429242, and the controls DMSO and tunicamycin were prespotted onto empty 384-well plates (Corning, Falcon 384-well Optilux Black/Clear Flat Bottom) using Labcyte Echo Acoustic dispensing (Beckman Coulter). The screening was performed with final concentrations of 10 µM for compounds, 10 µM of PF429242, and 0.1% DMSO and 2 µg mL^{-1} tunicamycin as controls. Prespotted plates were stored at –20 °C until the day of the experiment. HEK293-BiP-HT cells were incubated with MaP555 (100 nM) for 30 min in growth medium, then the cells were washed twice with 1× PBS and incubated in growth medium for 30 min. After washing twice with 1× PBS, the cells were trypsinized and resuspended in imaging medium (FluoroBrite DMEM, Gibco) supplemented with 10% FBS. The cell suspension was diluted with imaging medium and probe Sir-Br (100 nM) was added. Distribution of the cell suspension into the previously prepared plates (4'000 cells per well) was carried out using a Multidrop Combi Reagent Dispenser (Thermo Fisher) with a Standard Tube Plastic Tip Cassette (Steinle

Labtechnology). The plates were incubated at 25 °C for 15 min, then transferred to an incubator and incubated at 37 °C in a 5% CO_2 atmosphere.

Imaging and Analysis. Imaging was performed using a GE Healthcare IN Cell Analyzer 2200 (Nikon 10X/0.45 objective, Cy3 (ex 542/27, em 597/45) and Cy5 (ex 632/22, em 684/25) channels) after 6 and 24 h. A single field of view was acquired for each well, covering about 1/4 of the well surface. Before the first imaging session, the plates were covered with membranes (Breathe-Easy sealing membrane). Image analysis was performed using CellProfiler software (v4.2.5),[3] and results analysis and compilation were performed at the BSF using an in-house LIMS. The image analysis pipeline was composed of several subsequent steps. First, the images were preprocessed by removing the background using a Gaussian filter size of smoothing size 100. Then, corrected images were rescaled and smoothed to increase contrast and improve segmentation. Preprocessed images from both Cy3 and C5 channels were then added to create an intermediate image used for segmentation of all cells using 3-class Otsu thresholding. Intensity features were then extracted from all segmented cells for both channels. Three different read-out methods were used: the mean fluorescence intensity of cells (SiR-Br channel), the ratio of the mean fluorescence intensities of SiR-Br and MaP555, and the percentage of cells per field of view above a manually set threshold of fluorescence intensity of SiR-Br. For each readout, hits were identified and validated when the averaged normalized value of both replicates was greater than three times the standard deviation of the negative control replicates of the corresponding plate (negative = 0, positive = 1). For the classification of compounds as hits, we mainly relied on the read-out of the percentage of cells.

Determination of Z' factor. For validation of the assay, 384-well plates with the control conditions were prepared (first round: 1/2 plate DMSO (0.1%), 1/2 plate tunicamycin (2 µg mL^{-1}); second round: 1/6 plate DMSO (0.1%), 1/6 plate DMSO + PF-429242 (10 µM), 1/3 plate tunicamycin (2 µg mL^{-1}) + PF-429242 (10 µM), 1/3 plate tunicamycin (2 µg mL^{-1})). The Z' factors were calculated using the equation (3):[4]

$$Z' = 1 - \frac{(3\sigma_{c+} + 3\sigma_{c-})}{|\,\mu_{c+} - \mu_{c-}\,|} \qquad (3)$$

where c+ is the positive control and c– is the negative control. For the second round, the Z' factors were calculated based on tunicamycin + PF-429242 as the negative control and tunicamycin as the positive control. Each plate of the screen was also validated with a Z' calculated based on two columns of negative control replicates and

two columns of positive control replicates. Those control columns were also used for normalization of the results per plate, allowing inter-plate results comparisons.

11 Synthesis

The synthesis of SiR-Br was adapted from published procedures.[5,6] The synthesis of MaP555 is described in reference[6] and was performed by Zacharias Thiel. The synthesis of **S1** is described in reference[5] and was performed by Salome Püntener.
First, protection of the carboxylic acid groups of 2-bromoterephthalic acid (**S2**) was carried out. The amide **S3** was formed using thionyl chloride and 2-amino-2-methyl-1-propanol (**S4**). After cyclization, the dihydrooxazole compound **S5** was obtained. This compound was reacted with silicone-xanthone **S1** to form the rhodamine scaffold. Deprotection of rhodamine **S6** led to **S7,** and subsequent allyl protection gave **S8**. The cyanamide moiety was installed using oxalyl chloride, followed by cyanamide, resulting in the allyl-protected cyanamide compound **S9**. Deprotection of the carboxylic group gave **S10**. Finally, the bromoalkane HaloTag ligand **S11** was introduced after deprotection of its Boc-protected version **S12**, resulting in the compound SiR-Br **S13**.

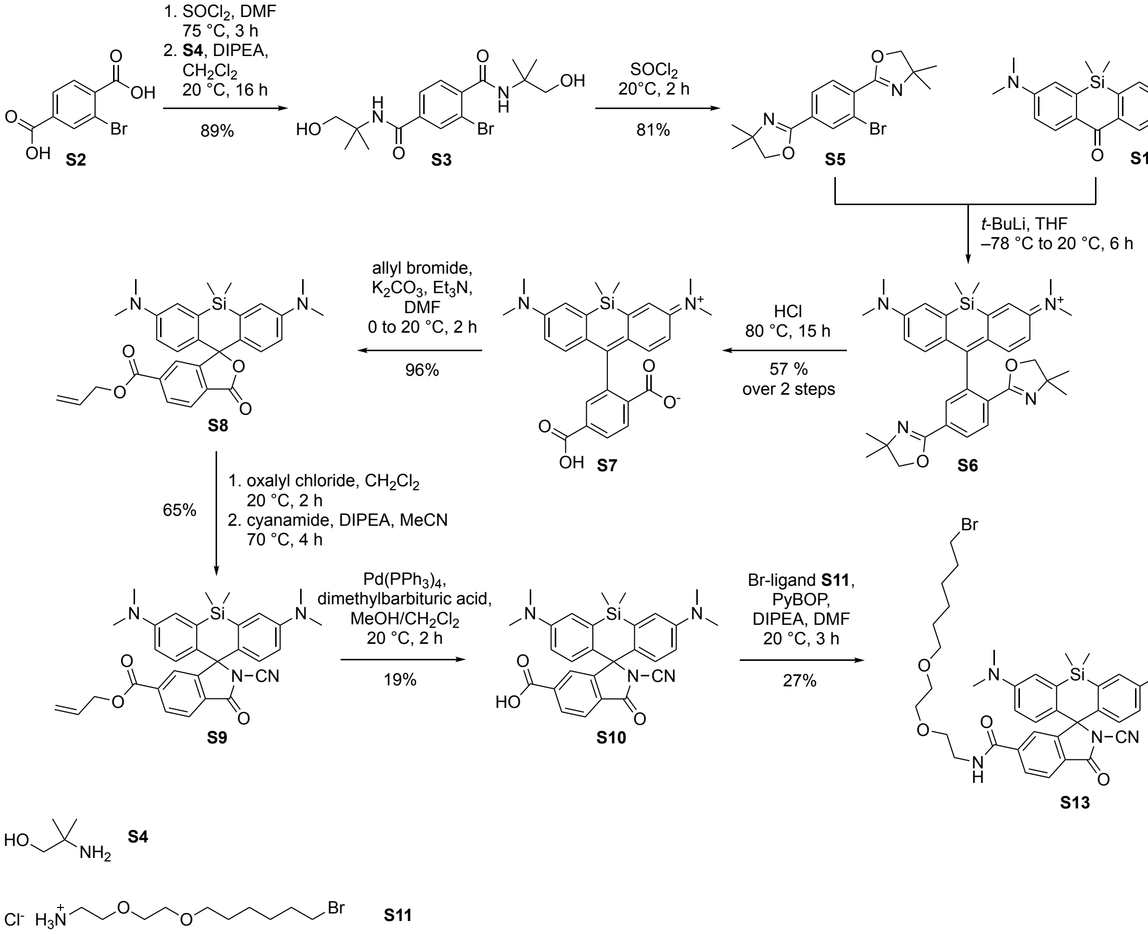

Supplementary Scheme 1. Synthesis of SiR-Br.

2-Bromo-N^1,N^4-bis(1-hydroxy-2-methylpropan-2-yl)terephthalamide (S3)

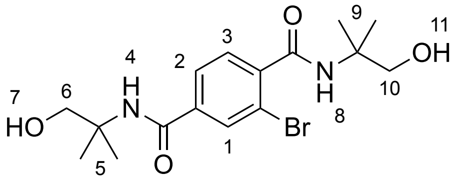

In a flame-dried flask under inert atmosphere, a suspension of 2-bromoterephthalic acid (1.0 g, 4.08 mmol, 1.0 equiv.) in thionyl chloride (6.7 mL) and dry DMF (5 µL, 1 drop) was stirred at 75 °C for 3 h. The reaction mixture was cooled to 20 °C and the solvent was evaporated under reduced pressure. The residue was dissolved in dry CH_2Cl_2 (13 mL) and added dropwise to a solution of 2-amino-2-methyl-1-propanol (980 mg, 11.0 mmol, 2.7 equiv.) and DIPEA (2 mL, 12.2 mmol, 3.0 equiv.) in dry CH_2Cl_2 (10 mL). The solution was stirred at 20 °C for 16 h, then saturated aqueous $NaHCO_3$ solution was added to the reaction mixture. After extraction with EtOAc, the combined organic phases were washed with water and brine, dried over Na_2SO_4, filtered, and evaporated under reduced pressure to obtain **S3** (1.41 g, 3.64 mmol, 89%) as a white fluffy powder. ^{1}H NMR (400 MHz, $(CD_3)_2SO$) δ = 8.03 (d, J = 1.6 Hz, 1H, H1), 7.84 (s, 1H,

H8), 7.80 (dd, J = 7.9, 1.6 Hz, 1H, H2), 7.71 (s, 1H, H4), 7.42 (d, J = 7.9Hz, 1H, H3), 4.85 (t, J = 6.0Hz, 1H, H7 or H11), 4.80 (t, J = 6.0 Hz, 1H, H7 or H11), 3.50 (t, J = 6.3 Hz, 4H, H6&H10), 1.30 (s, 6H, H5 or H9), 1.30 (s, 6H, H5 or H9) ppm. ^{13}C NMR (101 MHz, $(CD_3)_2SO$) δ = 166.7, 164.4, 141.8, 137.2, 131.1, 128.3, 126.4, 118.6, 67.3, 67.0, 55.3, 55.3, 23.5, 23.4 ppm. HRMS (ESI) calculated for $[C_{16}H_{23}BrN_2O_4+H]^+$: 387.0914, found 387.0919.

2,2'-(2-Bromo-1,4-phenylene)bis(4,4-dimethyl-4,5-dihydrooxazole) (S5)

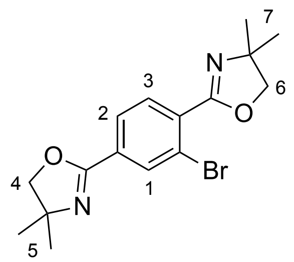

In a flame-dried flask under inert atmosphere, a solution of **S3** (730 mg, 1.88 mmol, 1.0 equiv.) in thionyl chloride (4.5 mL) was stirred at 20 ºC for 2 h. The solution was added dropwise to a saturated aqueous $NaHCO_3$ solution. The resulting water phase was extracted with EtOAc, then the combined organic phases were washed with water and brine, dried over Na_2SO_4, filtered, and evaporated. The residue was purified by flash column chromatography (SiO_2; CH_2Cl_2/EtOAc 9:1 to 3:2) to obtain **S5** (537 mg, 1.53 mmol, 81%) as a white solid. ^{1}H NMR (400 MHz, $(CD_3)_2SO$) δ = 8.07 (d, J = 1.5 Hz, 1H, H1), 7.90 (dd, J = 8.0, 1.6 Hz, 1H, H2), 7.75 (d, J = 8.0 Hz, 1H, H3), 4.15 (s, 2H, H4), 4.12 (s, 2H, H6), 1.31 (s, 6H, H5 or H7), 1.29 (s, 6H, H5 or H7) ppm. ^{13}C NMR (101 MHz, $(CD_3)_2SO$) δ = 159.6, 158.8, 132.2, 132.2, 131.6, 130.8, 126.8, 120.9, 78.8, 78.6, 68.1, 67.7, 28.1, 27.9 ppm. HRMS (ESI) calculated for $[C_{16}H_{19}BrN_2O_2+H]^+$: 351.0703, found 351.0704.

***N*-(10-(2,5-Bis(4,4-dimethyl-4,5-dihydrooxazol-2-yl)phenyl)-7-(dimethylamino)-5,5-dimethyldibenzo[*b*,*e*]silin-3(5*H*)-ylidene)-*N*-methylmethanaminium (S6)**

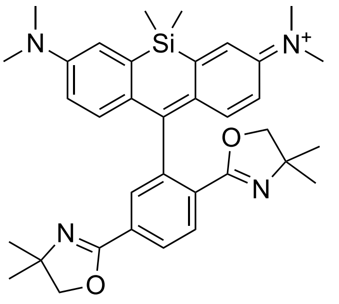

In a three-neck flask equipped with a thermometer under inert atmosphere, a solution of **S5** (500 mg, 1.42 mmol, 3.0 equiv.) in dry THF (26 mL) was cooled to –78 °C, and *tert*-butyllithium (1.7 M, 0.85 mL, 1.42 mmol, 3.0 equiv.) was added dropwise at the same temperature. The yellow, then orange solution was stirred at –78 °C for an additional 30 min. Next, **S1** (154 mg, 0.48 mmol, 1.0 equiv.) in dry THF (14 mL) was added dropwise at –78 °C, then the orange solution was warmed up to 20 ºC and stirred at 20 °C for 6 h. The reaction mixture was cooled to 0 ºC and acetic acid (2.6 mL) was added to neutralize *t*-BuLi. The resulting intense blue solution was concentrated under

reduced pressure and the crude product **S6** was directly used in the subsequent reaction without further purification.

4-Carboxy-2-(7-(dimethylamino)-3-(dimethyliminio)-5,5-dimethyl-3,5-dihydrodibenzo[*b,e*]silin-10-yl)benzoate (S7)

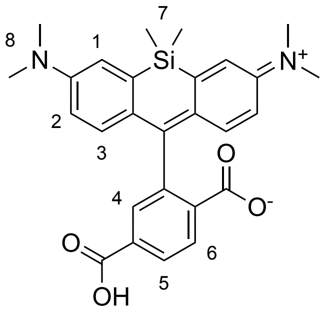

S6 (275 mg, 0.48 mmol, 1.0 equiv.) was dissolved in hydrochloric acid solution (6 M, 55 mL) and stirred at 80 °C for 15 h. After cooling to 20 ºC, the solution was added to a saturated aqueous $NaHCO_3$ solution. The resulting mixture was extracted with CH_2Cl_2, and the combined organic layers were washed with aqueous HCl solution (0.1 M) and brine, dried over Na_2SO_4, filtered, and evaporated. The crude product was purified by flash chromatography (SiO_2; CH_2Cl_2 to CH_2Cl_2/MeOH 95:5) to obtain product **S7** as a blue solid (127 mg, 0.27 mmol, 57%). ^{1}H NMR (400 MHz, $CDCl_3$) δ = 8.23 (dd, *J* = 8.0, 1.3 Hz, 1H, H5), 8.03 (dd, *J* = 8.0, 0.8 Hz, 1H, H6), 7.99 (t, *J* = 1.0 Hz, 1H, H4), 6.97 (d, *J* = 2.9 Hz, 2H, H1), 6.79 (d, *J* = 8.9 Hz, 2H, H3), 6.57 (dd, *J* = 9.0, 2.9 Hz, 2H, H2), 2.97 (s, 12H, H8), 0.67 (s, 3H, H7), 0.60 (s, 3H, H7). HRMS (ESI) calculated for $[C_{27}H_{28}N_2O_4Si{-}H]^-$: 471.1746, found 471.1764.

Allyl 3,7-bis(dimethylamino)-5,5-dimethyl-3'-oxo-3'*H*,5*H*-spiro[dibenzo[*b,e*]siline-10,1'-isobenzofuran]-6'-carboxylate (S8)

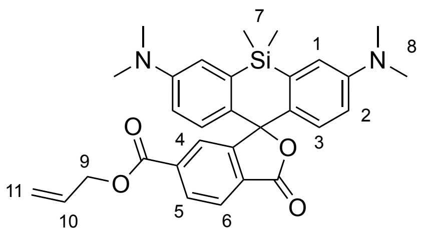

In a flame-dried flask under an inert atmosphere, **S7** (65 mg, 0.14 mmol, 1.0 equiv.) was dissolved in dry DMF (2.0 mL). After addition of potassium carbonate (39 mg, 0.28 mmol, 2.0 equiv.) and NEt_3 (39 µL, 0.28 mmol, 2.0 equiv.), the reaction was cooled to 0 ºC, and allyl bromide (19 µL, 0.22 mmol, 1.5 equiv.) was slowly added. The reaction mixture was warmed to 20 °C and stirred for 2 h. The mixture was diluted with water and extracted with CH_2Cl_2, then the combined organic layers were washed with brine, dried over Na_2SO_4, filtered, and concentrated. The product **S8** was obtained without further purification (68 mg, 0.13 mmol, 96%). ^{1}H NMR (400 MHz, $CDCl_3$) δ = 8.20 (dd, *J* = 8.0, 1.3 Hz, 1H, H5), 8.01 (dd, *J* = 8.0, 0.8 Hz, 1H, H6), 7.96 (dd, *J* = 1.3, 0.8 Hz, 1H, H4), 6.97 (d, *J* = 2.9 Hz, 2H, H1), 6.80 (d, *J* = 8.9 Hz, 2H, H3), 6.57 (dd, *J* = 8.9, 2.9 Hz, 2H, H2), 5.98 (ddt, *J* = 17.2, 10.4, 5.9 Hz, 1H, H10), 5.42 – 5.25 (m, 2H, H11),

4.78 (dt, J = 5.9, 1.4 Hz, 2H, H9), 2.97 (s, 12H, H8), 0.68 (s, 3H, H7), 0.60 (s, 3H, H7) ppm.

Allyl 2'-cyano-3,7-bis(dimethylamino)-5,5-dimethyl-3'-oxo-5*H*-spiro[dibenzo[*b*,*e*]siline-10,1'-isoindoline]-6'-carboxylate (S9)

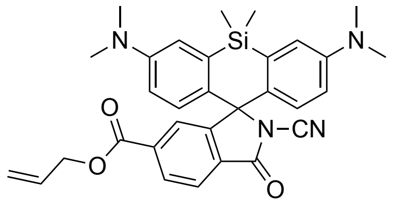

In a flame-dried flask under inert atmosphere, a solution of **S8** (68 mg, 0.13 mmol, 1.0 equiv.) in dry CH_2Cl_2 (12 mL) was cooled to 0 ºC, then oxalyl chloride (17 µL, 0.20 mmol, 1.5 equiv.) was added dropwise, and the dark blue solution was stirred at 20 ºC for 2 h. The volatiles were removed under reduced pressure, and the crude residue was used in the subsequent reaction without further purification. A solution of cyanamide (66 mg, 1.57 mmol, 10 equiv.) and DIPEA (0.33 mL, 1.97 mmol, 15 equiv.) in dry CH_3CN (5.6 mL) was added to the acyl chloride residue. Dry CH_3CN (11.2 mL) was added quickly to the reaction and the reaction mixture was stirred at 70 °C for 4 h. The solvent was evaporated, and the residue was purified by column chromatography (SiO_2; CH_2Cl_2 to CH_2Cl_2/MeOH 95:5) to give the product **S9** as a green solid (46 mg, 0.09 mmol, 65%). The product was directly used in the next step.

2'-Cyano-3,7-bis(dimethylamino)-5,5-dimethyl-3'-oxo-5*H*-spiro[dibenzo[*b*,*e*]siline-10,1'-isoindoline]-6'-carboxylic acid (S10)

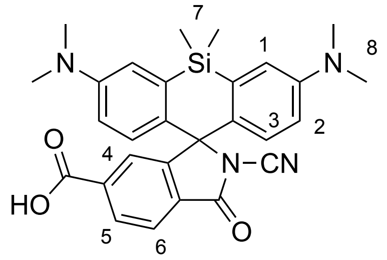

In a flame-dried flask under inert atmosphere, a solution of **S9** (45 mg, 0.84 mmol, 1.0 equiv.), 1,3-dimethyl-1,3-diazinane-2,4,6-trione (39 mg, 0.25 mmol, 3.0 equiv.) and tetrakis(triphenylphosphine)palladium(0) (48 mg, 0.04 mmol, 0.5 equiv.) in MeOH/CH_2Cl_2 (8.6 mL, 5:1 v/v) was stirred at 20 °C for 2 h. The solution was diluted with CH_2Cl_2, washed with saturated aqueous Na_2CO_3 solution, and extracted with CH_2Cl_2. The combined organic layers were dried over Na_2SO_4, filtered, and concentrated under reduced pressure. The residue was purified by reversed-phase chromatography (C18; H_2O/CH_3CN 9:1 to CH_3CN) to yield **S10** (8 mg, 0.16 mmol, 19%). ^{1}H NMR (400 MHz, $CDCl_3$) δ = 8.16 (dd, J = 8.1, 1.3 Hz, 1H, H5), 8.06 (d, J = 8.0 Hz, 1H, H6), 7.56 (s, 1H, H4), 7.01 (d, J = 2.6 Hz, 2H, H1), 6.82 – 6.67 (m, 4H, H2&H3), 2.99 (s, 12H, H8), 0.65 (s, 3H, H7), 0.60 (s, 3H, H7) ppm.

***tert*-Butyl (2-(2-((6-bromohexyl)oxy)ethoxy)ethyl)carbamate (S12)**

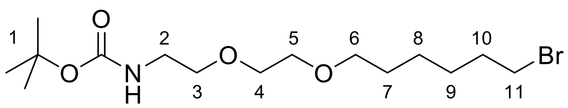

In a flame-dried flask under inert atmosphere, sodium hydride (60%, 585 mg, 14.6 mmol, 3.0 equiv.) was added to a solution of 2-(2-boc-aminoethoxy)-ethanol (1.0 g, 4.87 mmol, 1.0 equiv.) and 1,6-dibromohexane (2.3 mL, 14.6 mmol, 3.0 equiv.) in dry THF (10 mL) at 0–5 °C. The reaction was stirred at 20 °C for 24 h, then poured into water (10 mL). The reaction mixture was extracted with EtOAc, then the combined organic layers were washed with brine, dried over Na_2SO_4, and concentrated. The crude product was purified by flash column chromatography (SiO_2; hexane to hexane/EtOAc 3:1), which gave product **S12** as a colorless oil (370 mg, 1.0 mmol, 21%). ^{1}H NMR (400 MHz, $CDCl_3$) δ = 3.63–3.59 (m, 2H, H3, H4 or H5), 3.58–3.52 (m, 4H, H3, H4 or H5), 3.46 (t, J = 6.6 Hz, 2H, H6), 3.41 (t, J = 6.8 Hz, 2H, H11), 3.31 (s, 2H, H2), 1.91–1.82 (m, 2H, H10), 1.65–1.58 (m, 2H, H7), 1.49–1.35 (m, 13H, H1,H8,H9) ppm. ^{13}C NMR (101 MHz, $CDCl_3$) δ = 71.4, 70.4, 70.4, 70.2, 34.0, 32.9, 29.6, 28.6, 28.1, 25.5 ppm. HRMS (ESI) calculated for $[C_{15}H_{30}BrNO_4Si+Na]^+$: 390.1250, found 390.1254.

***N*-(2-(2-((6-bromohexyl)oxy)ethoxy)ethyl)-2'-cyano-3,7-bis(dimethylamino)-5,5-dimethyl-3'-oxo-5*H*-spiro[dibenzo[*b*,*e*]siline-10,1'-isoindoline]-6'-carboxamide (S13)**

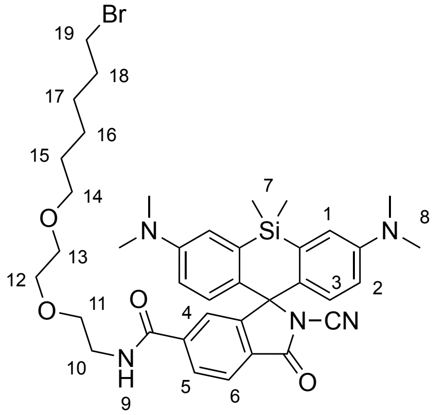

A solution of the protected ligand **S12** (17 mg, 48 µmol, 3.0 equiv.) in HCl in dioxane (4 M, 3 mL) and stirred at 60 °C for 24 h. The solvent was evaporated under reduced pressure and the resulting solid was washed with diethyl ether to yield the deprotected Br-ligand. The crude product was used without further purification in the next step. In a flame-dried flask under inert atmosphere, Br-ligand **S11** (13 mg, 48 µmol, 3.0 equiv.), **S10** (8 mg, 16 µmol, 1.0 equiv.) and PYBOP (17 mg, 33 µmol, 2.0 equiv.) were combined in dry DMF (0.8 mL). DIPEA (30 µL, 0.18 mmol, 11 equiv.) was added and the resulting solution was stirred at 20 ºC for 5 h. All volatiles were removed and the residue was purified by preparative TLC (SiO_2; EtOAc/hexane 7:3) to yield **S13** as a pale-yellow solid (3.3 mg, 4 µmol, 27%). ^{1}H NMR (800 MHz,

$(CD_3)_2SO$) δ = 8.76 (t, *J* = 5.6 Hz, 1H, H9), 8.09 (d, *J* = 8.1 Hz, 1H, H6), 8.02 (dd, *J* = 8.1, 1.4 Hz, 1H, H5), 7.36 (d, *J* = 1.4 Hz, 1H, H4), 6.98 (d, *J* = 2.9 Hz, 2H, H1), 6.73 (dd, *J* = 9.1, 2.8 Hz, 2H, H3), 6.66 (d, *J* = 9.1 Hz, 2H, H2), 3.58 (t, *J* = 6.6 Hz, 2H, H19), 3.50–3.39 (m, 6H, H10–14), 3.30–3.26 (m, 2H, H10–14), 2.94 (s, 12H, H8), 1.65 (dt, *J* = 14.6, 6.7 Hz, 2H, H18), 1.44–1.36 (m, 2H, H15), 1.35–1.29 (m, 2H, H17), 1.28–1.21 (m, 2H, H16), 0.62 (s, 3H, H7), 0.55 (s, 3H, H7) ppm. ^{13}C NMR (201 MHz, $(CD_3)_2SO$) δ = 167.3, 165.0, 155.5, 149.6, 141.7, 135.9, 129.0, 128.4, 127.9, 126.7, 125.5, 123.1, 116.2, 115.6, 107.4, 75.0, 70.6, 70.0, 69.8, 69.1, 45.8, 35.6, 32.6, 32.5, 29.5, 29.4, 27.8, 26.5, 25.3, 25.2, 0.6, 0.3 ppm. HRMS (ESI) calculated for $[C_{38}H_{48}BrN_5O_4Si+H]^+$: 746.2732; Found 746.2735.

11 NMR spectra

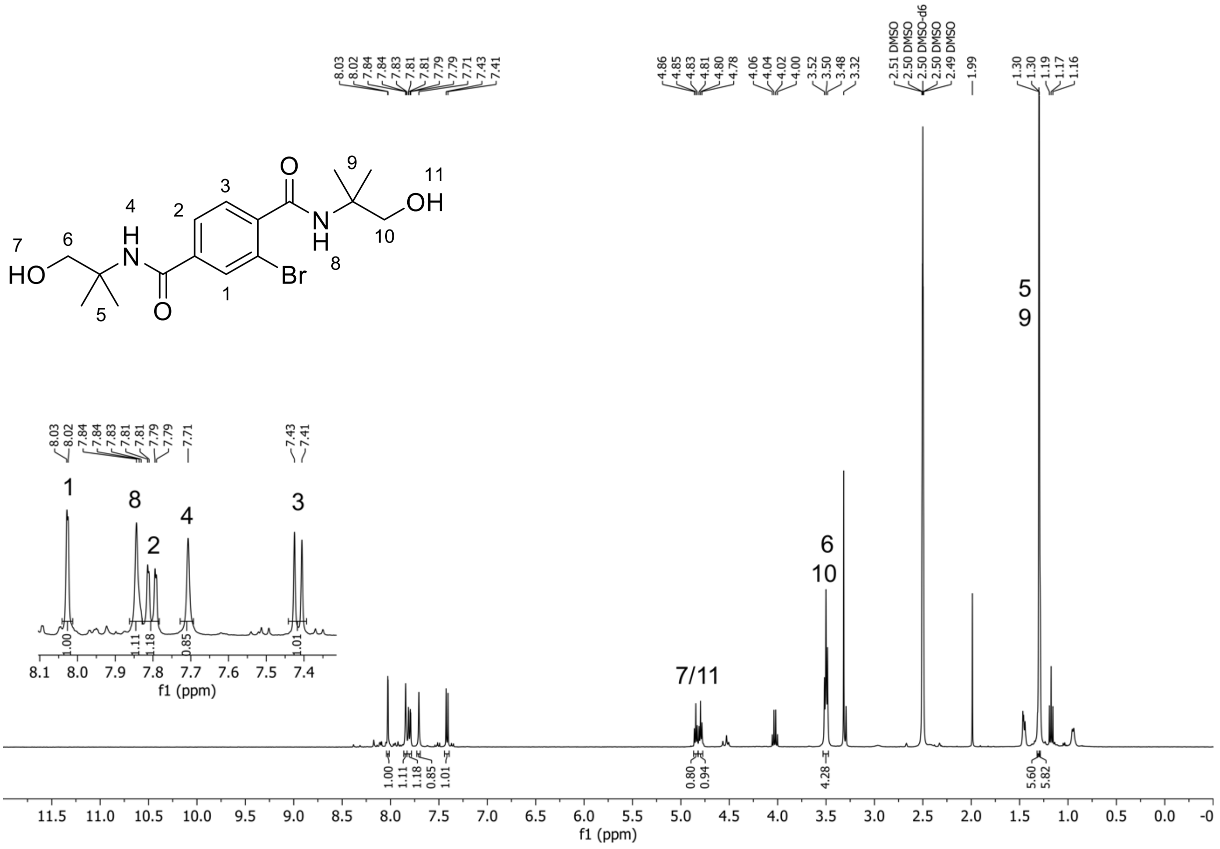

^1H NMR (400 MHz, $(CD_3)_2SO$) spectrum of compound **S3**.

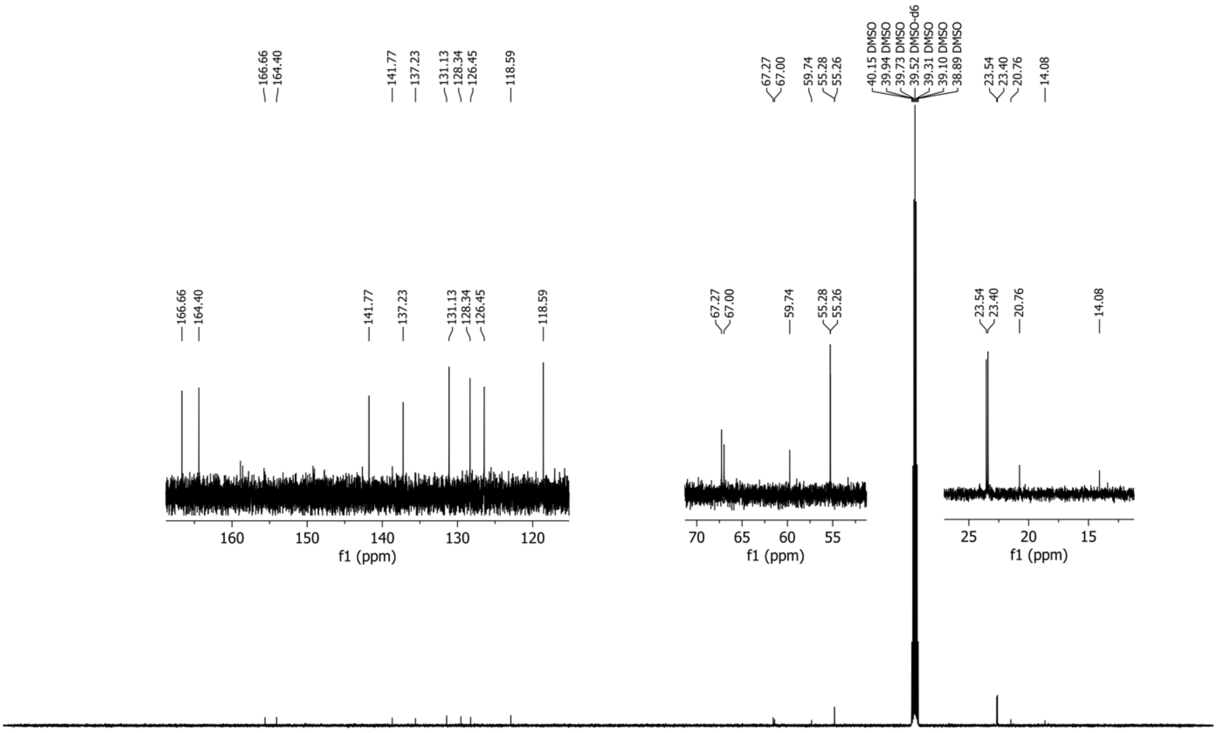

^{13}C NMR (101 MHz, $(CD_3)_2SO$) spectrum of compound **S3**.

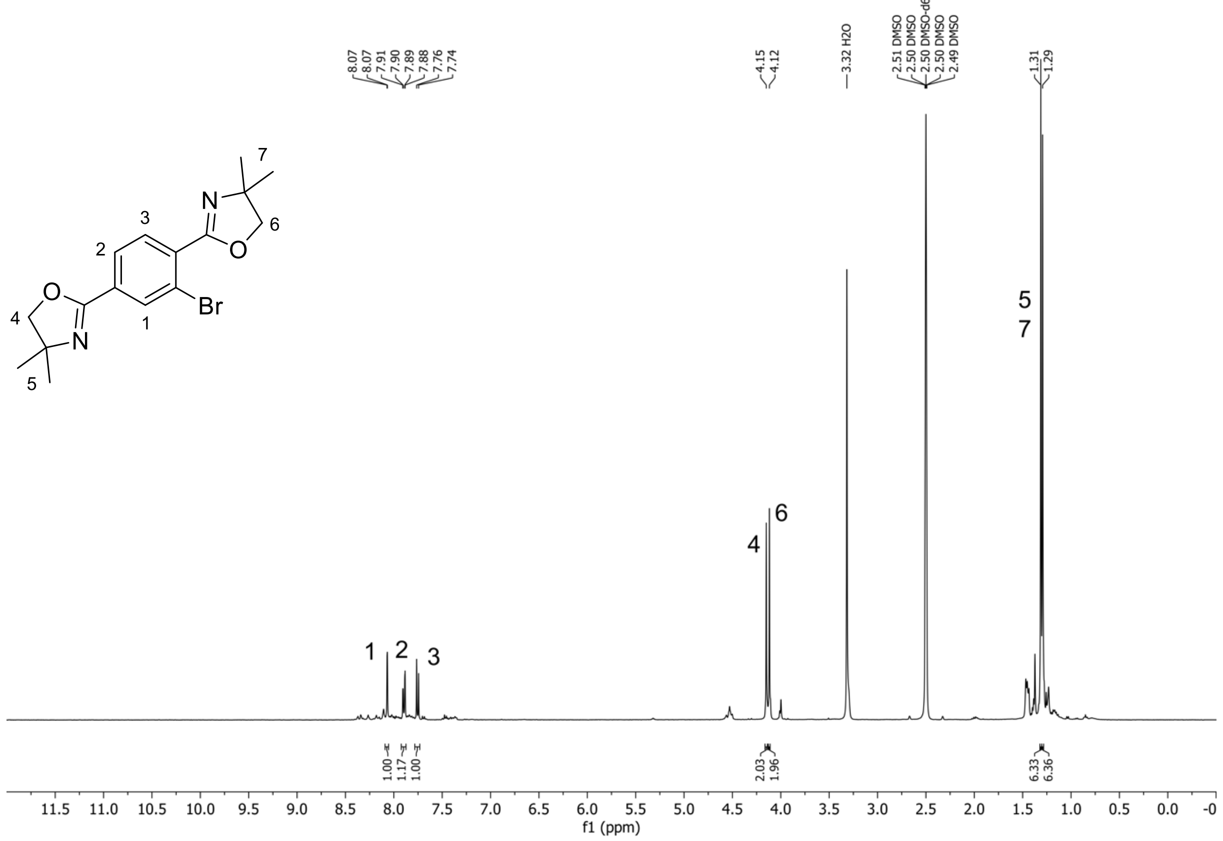

^{1}H NMR (400 MHz, $(CD_3)_2SO$) spectrum of compound **S5**.

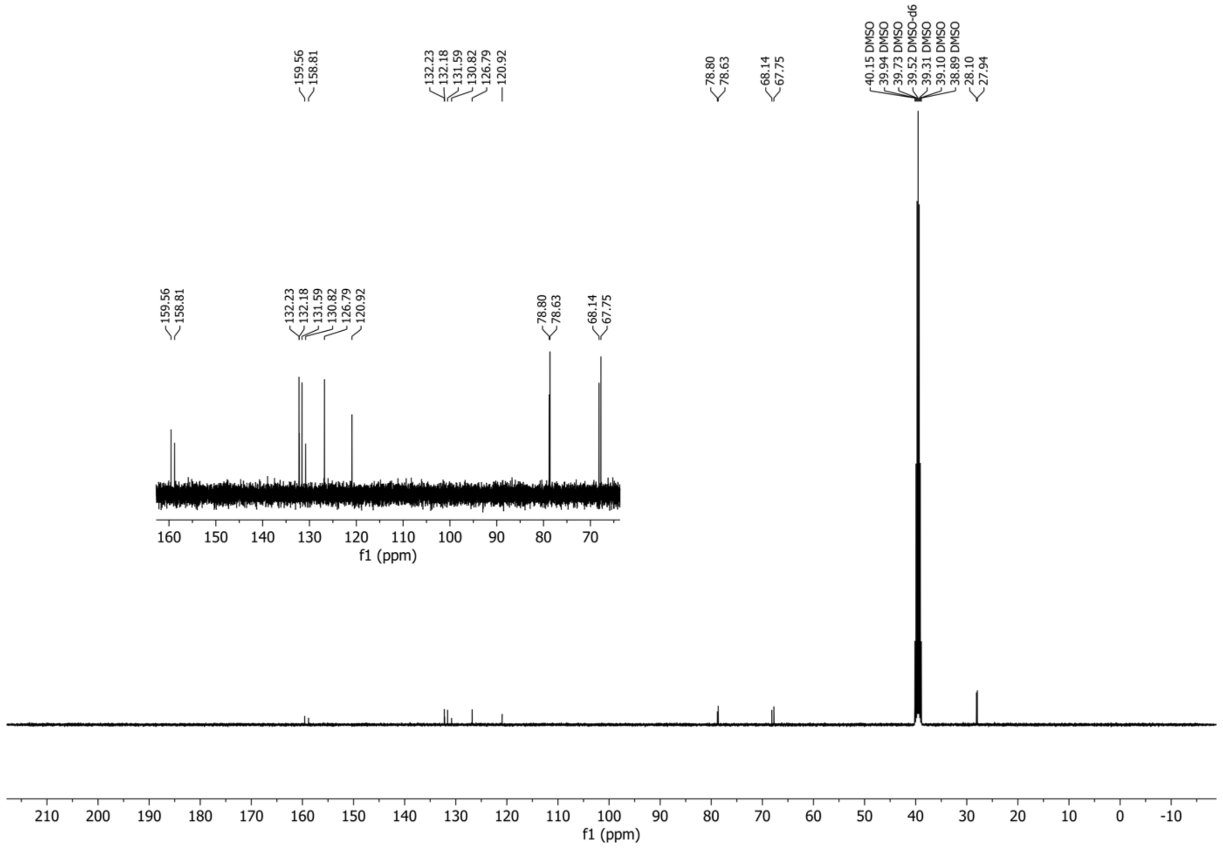

^{13}C NMR (101 MHz, $(CD_3)_2SO$) spectrum of compound **S5**.

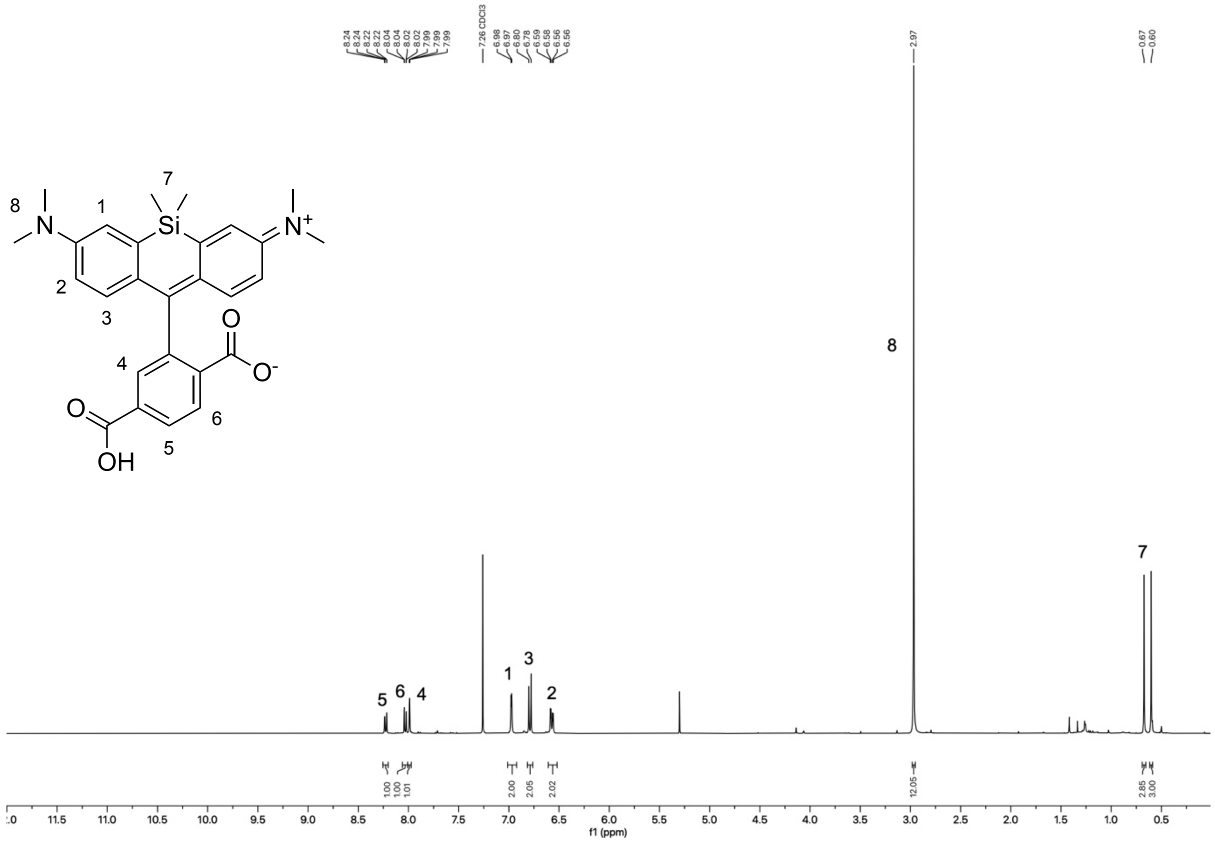

^{1}H NMR (400 MHz, $CDCl_3$) spectrum of compound **S7**.

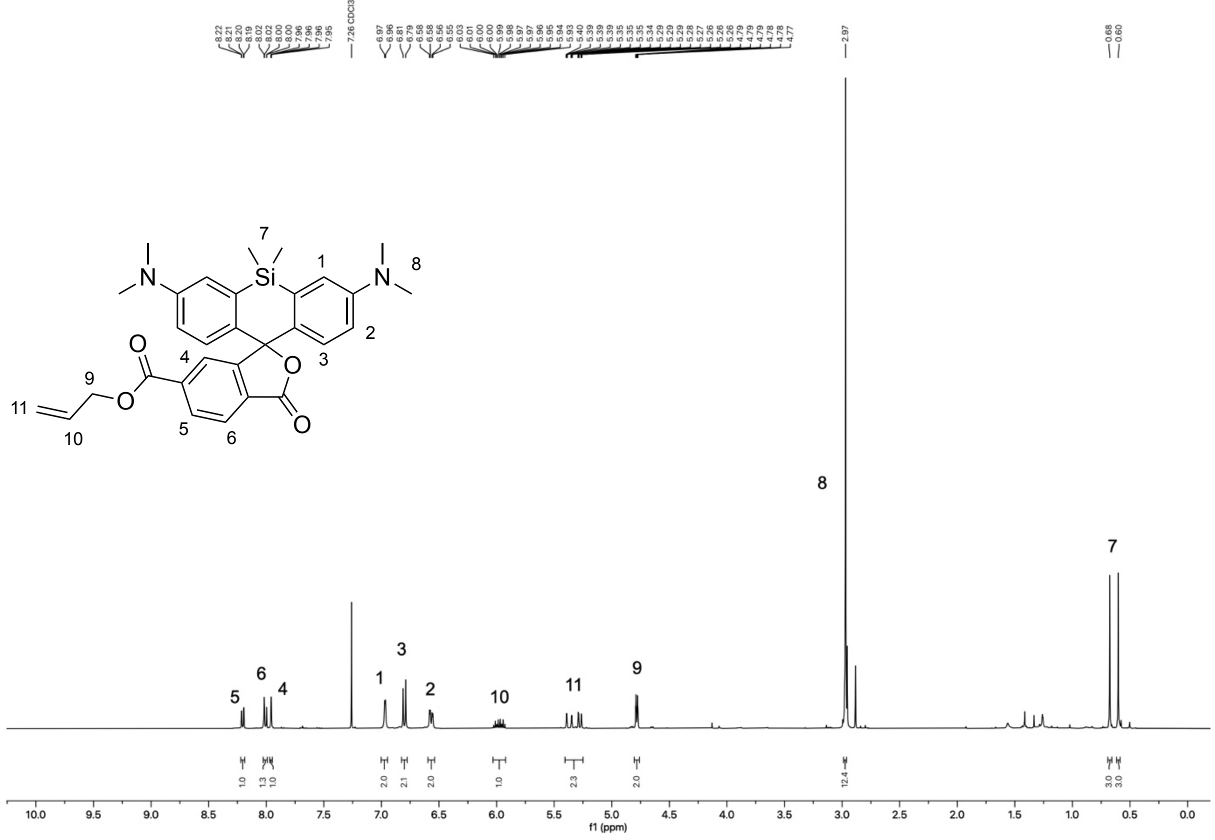

^{1}H NMR (400 MHz, $CDCl_3$) spectrum of compound **S8**.

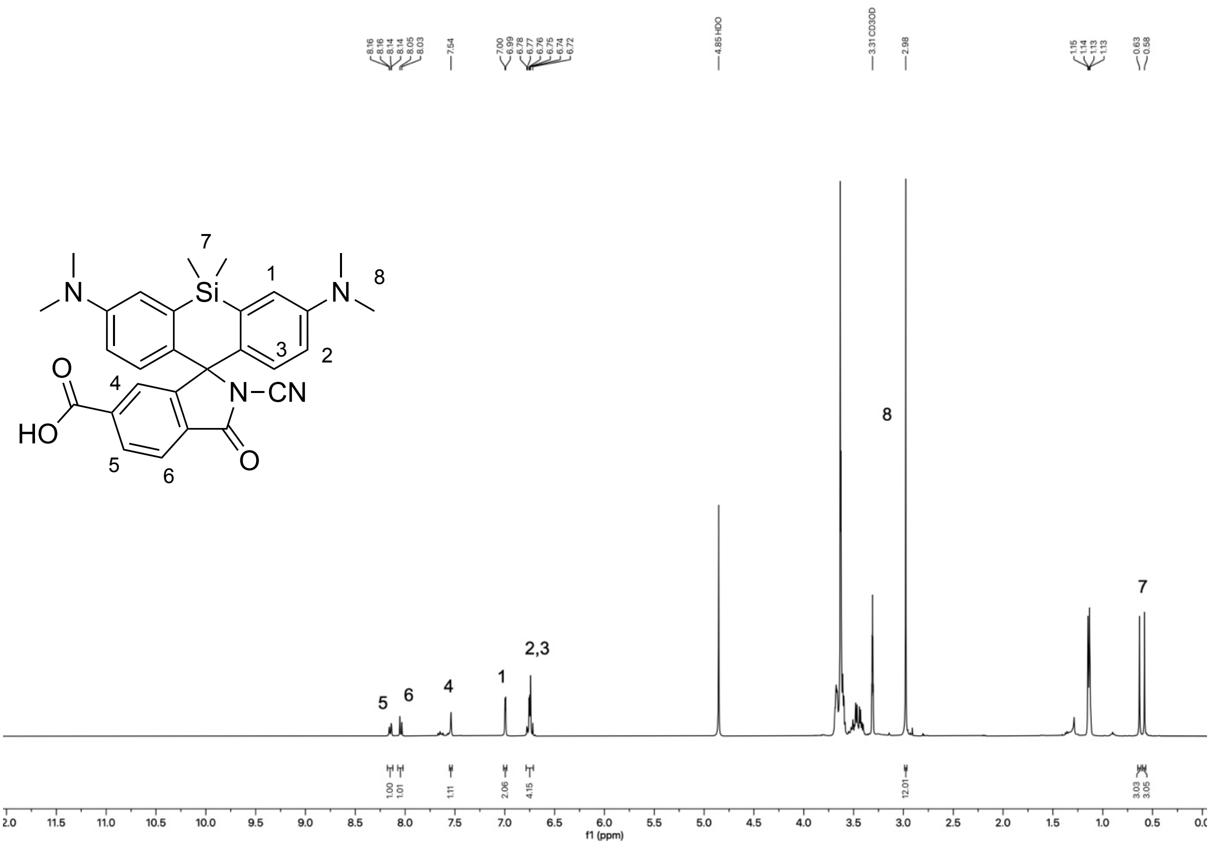

^{1}H NMR (400 MHz, CD_3OD) spectrum of compound **S10**.

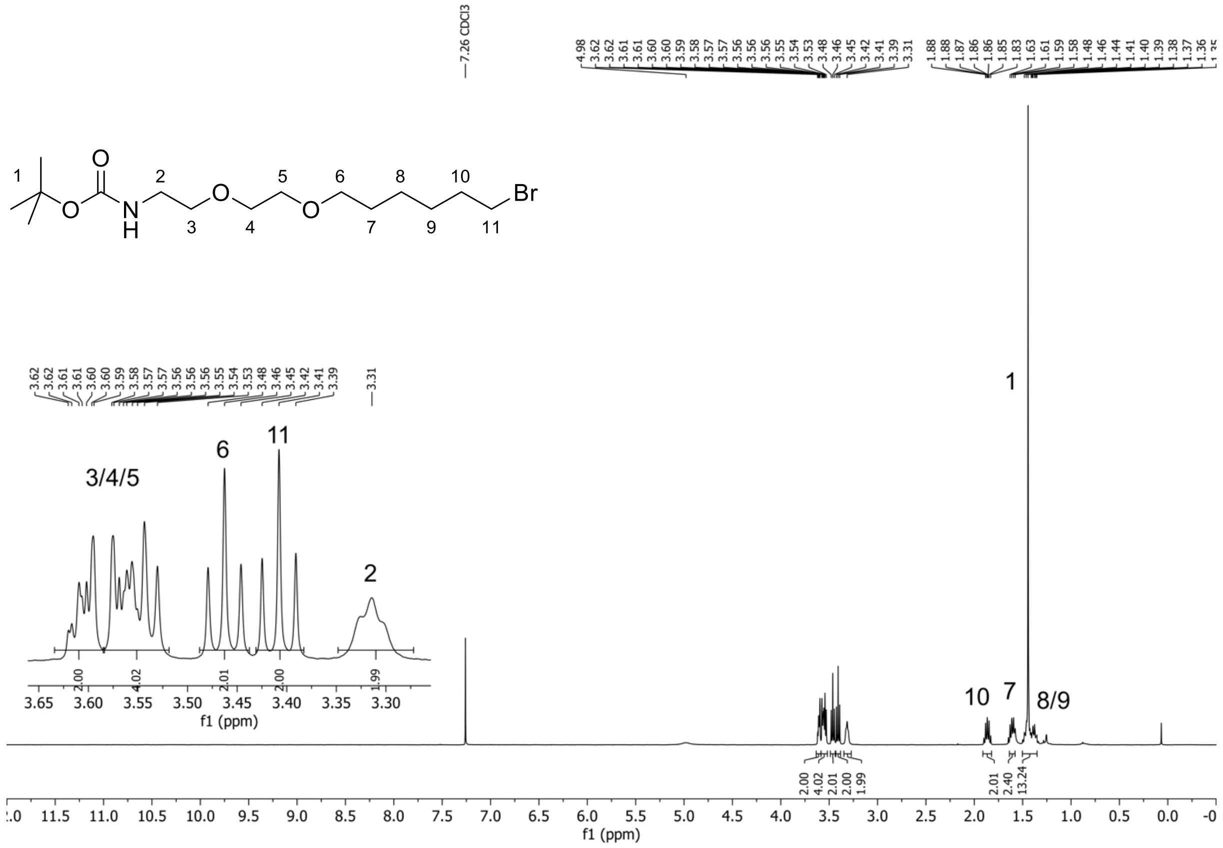

^{1}H NMR (400 MHz, $CDCl_3$) spectrum of compound **S12**.

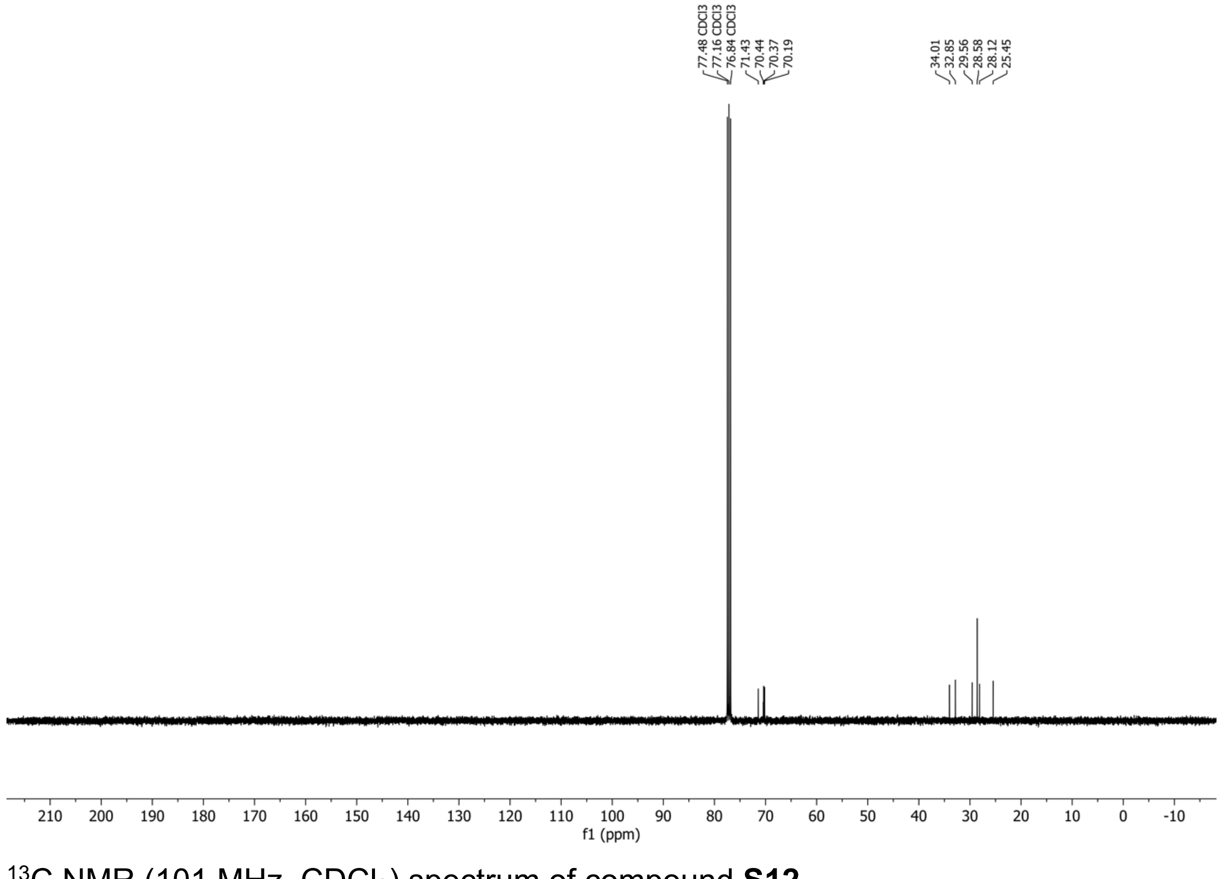

^{13}C NMR (101 MHz, $CDCl_3$) spectrum of compound **S12**.

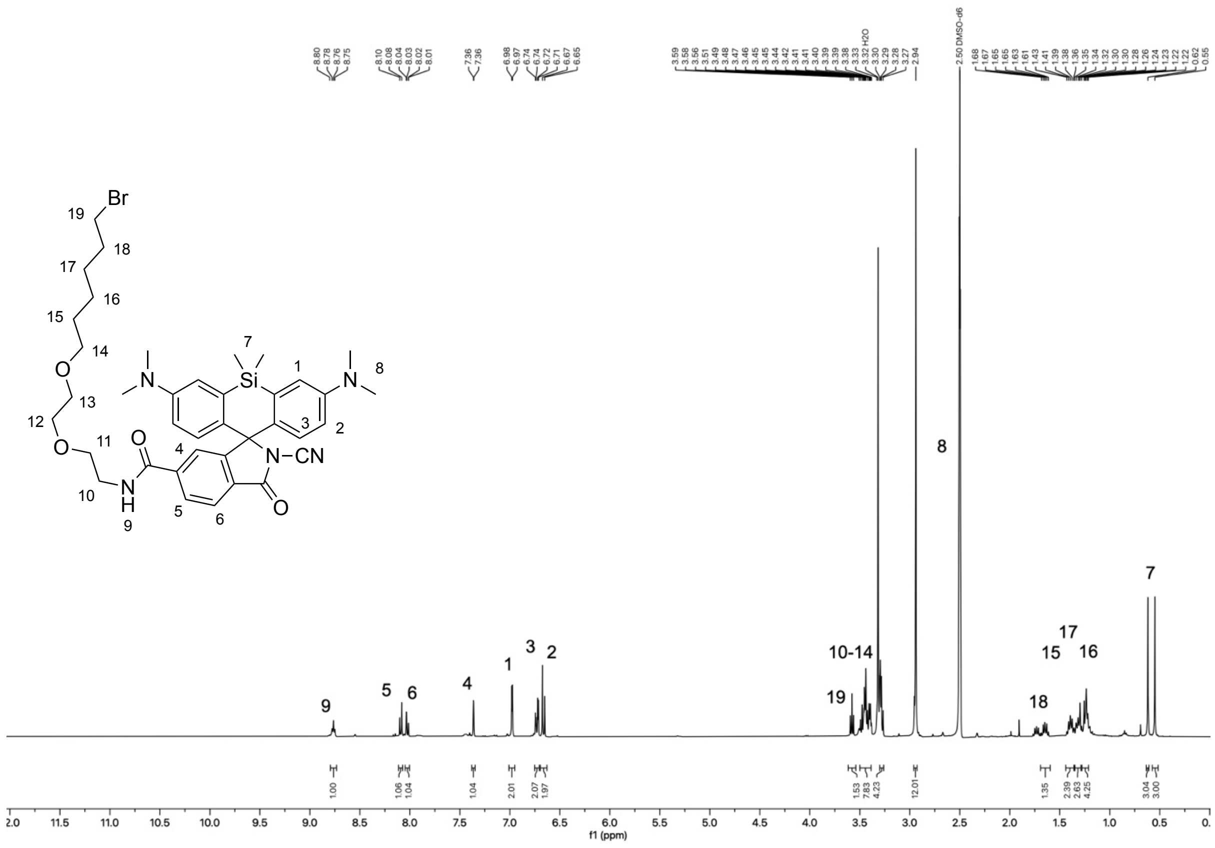

^{1}H NMR (800 MHz, $(CD_3)_2SO$) spectrum of compound **S13**.

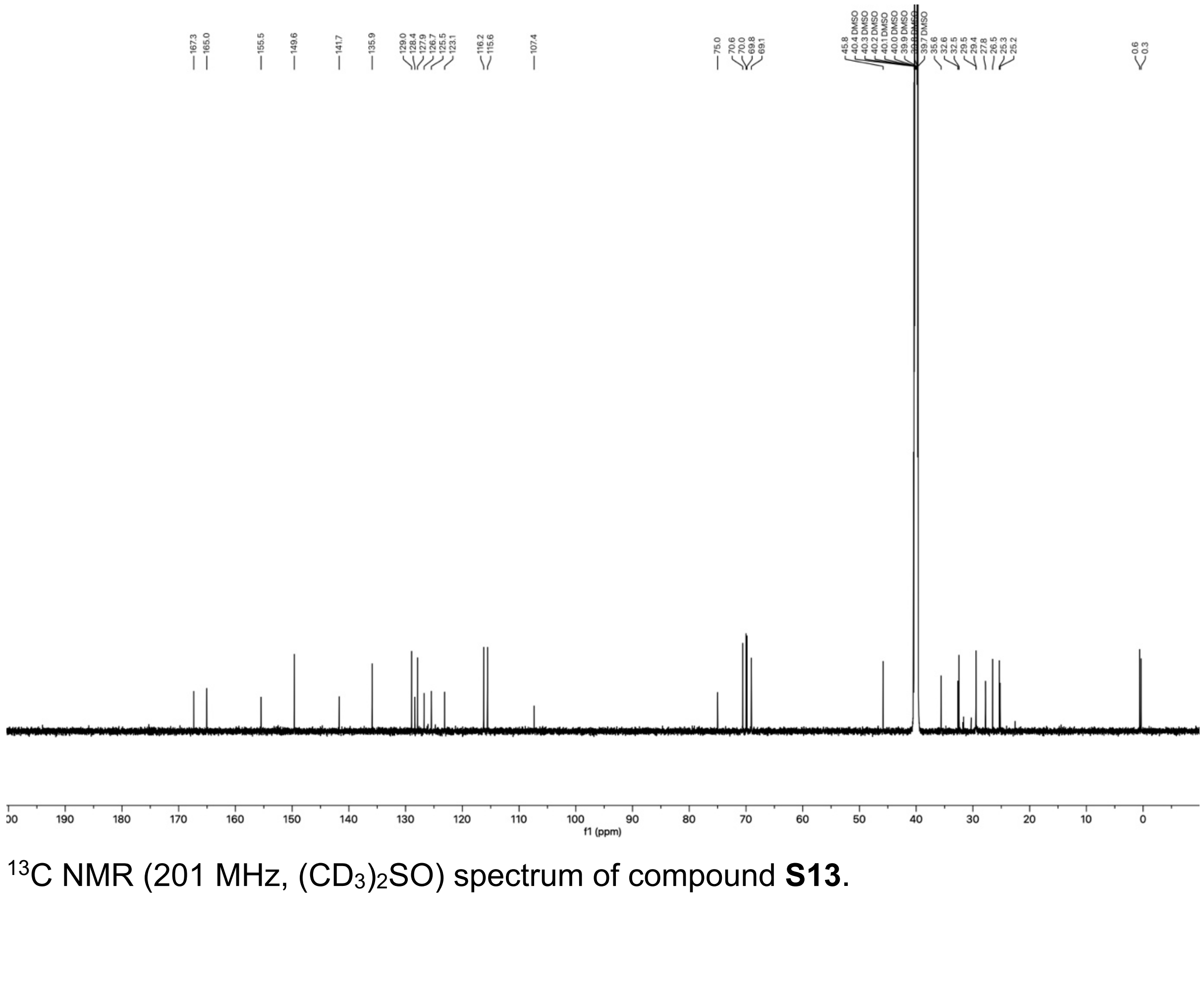

^{13}C NMR (201 MHz, $(CD_3)_2SO$) spectrum of compound **S13**.

12 Supplementary References

[1] T. Sakuma, S. Nakade, Y. Sakane, K.-I. T. Suzuki, T. Yamamoto, *Nat. Protoc.* **2016**, *11*, 118–133.

[2] Pfaffl, Michael W., in *Real-Time PCR*, Taylor & Francis, London, **2006**.

[3] D. R. Stirling, M. J. Swain-Bowden, A. M. Lucas, A. E. Carpenter, B. A. Cimini, A. Goodman, *BMC Bioinf.* **2021**, *22*, 433.

[4] J.-H. Zhang, T. D. Y. Chung, K. R. Oldenburg, *J. Biomol. Screen.* **1999**, *4*, 67–73.

[5] G. Lukinavičius, K. Umezawa, N. Olivier, A. Honigmann, G. Yang, T. Plass, V. Mueller, L. Reymond, I. R. Corrêa Jr, Z.-G. Luo, C. Schultz, E. A. Lemke, P. Heppenstall, C. Eggeling, S. Manley, K. Johnsson, *Nat. Chem.* **2013**, *5*, 132–139.

[6] L. Wang, M. Tran, E. D'Este, J. Roberti, B. Koch, L. Xue, K. Johnsson, *Nat. Chem.* **2020**, *12*, 165–172.
